# Invasive Alien Species of European Union Concern: A Systematic Review of High-Priority Pathogens in 22 Species in a One Health Framework

**DOI:** 10.3390/ani16091303

**Published:** 2026-04-23

**Authors:** Luca Spadotto, Cinzia Centelleghe, Luca Ceolotto, Sandro Mazzariol, Laura Cavicchioli

**Affiliations:** Department of Comparative Biomedicine and Food Science, University of Padua, 35020 Legnaro, Italy; cinzia.centelleghe@unipd.it (C.C.); luca.ceolotto@unipd.it (L.C.); sandro.mazzariol@unipd.it (S.M.); laura.cavicchioli@unipd.it (L.C.)

**Keywords:** emerging infectious diseases, European Union, IAS, One Health, pathogens, risk assessment, zoonotic pathogens

## Abstract

Invasive alien species (IAS) not only threaten biodiversity and ecosystems but also play a significant role in the spread of infectious diseases; however, the epidemiological role of many IAS remains poorly understood. This study presents the first systematic review of major pathogens reported in 22 IAS of concern to the European Community. Given the breadth of available data, we relied on a literature search including studies reporting natural infections in target IAS, excluding experimental infections and non-target species. A total of 541 publications between 1963 and 2023 were analyzed, identifying 472 pathogens, of which 64 were classified as high-priority based on key global and European frameworks. IAS with broader distribution and higher research effort were associated with greater pathogen richness, suggesting potential epidemiological relevance but also highlighting detection bias. A composite Host–Pathogen Influence Index (HPI-IAS) revealed spatial heterogeneity in epidemiological pressure across Europe, with Poland, Germany, Italy, and France identified as areas of elevated epidemiological concern. These findings underscore the urgent need for coordinated, cross-border monitoring strategies at the European level and contribute to a broader understanding of IAS-related infectious disease ecology within a One Health framework.

## Simple Summary

Invasive alien species are animals and plants introduced, intentionally or accidentally, outside their natural range, often with harmful consequences for local ecosystems and native animals. Beyond their ecological impact, these species can also carry and spread dangerous infectious diseases to humans, livestock, and wildlife. Despite growing concern, the full extent of this threat in Europe remains poorly understood. In this study, we reviewed the scientific literature to identify which microorganisms, including bacteria, viruses, fungi, parasites, and protozoa, are carried by 22 invasive species officially recognized as a concern by the European Union. We identified 64 pathogens of high public health relevance hosted by invasive mammals, birds, reptiles, and amphibians present across Europe. Several species, including the raccoon, the nutria, the muskrat, the gray squirrel, the raccoon dog, and the red-eared slider turtle, were found to carry particularly high numbers of dangerous pathogens capable of infecting humans. Our analysis also revealed that disease surveillance related to invasive species is uneven across Europe, with some countries, such as Poland, Germany, Italy, and France, representing higher-risk areas. These findings highlight the importance of coordinated, Europe-wide monitoring programs to better protect human and animal health.

## 1. Introduction

Alien or non-native species are all the animals, plants, fungi, and microorganisms expanding beyond their natural range and whose presence in a habitat is directly attributable to human activities [1]. Historically, humans have, unintentionally or not, used various ways to introduce new species into a new area. Specifically, animals have been relocated intentionally for many aims, such as keeping them as pets, as working animals, livestock, or other forms of production. Likewise, non-native plants are widely cultivated for food, feed, or ornamental purposes [2]. It is even possible that human actions can inadvertently translocate an alien species into a new territory, as exemplified by the introduction in Italy, via tire trade, of the Asian tiger mosquito (*Aedes albopictus)* [3,4], a competent vector of multiple arboviruses, increasingly recognized as a key driver of the emergence and spread of vector-borne diseases [5]. Once established in a new environment and under favorable conditions (e.g., rising levels of atmospheric CO2, along with increases in temperature and precipitation), such as those provided by climate change and anthropogenic exploitation of natural resources [6,7,8], some alien species can become “invasive” [9,10]. Notably, invasive alien species (IAS) can spread and flourish, usually exerting significant impacts on local ecosystems, economies, or human and animal health. The main characteristics of IAS are rapid spreading capability (e.g., dispersal capacity and range expansion speed), high reproductive output and growth, and broad adaptability to new environments, including wide thermal tolerance, dietary generalisms, and habitat flexibility [11,12,13,14], thus, these species may compete with and overcome the native ones, leading to community disruption and biodiversity loss [8].

As a result, the United Nations (UN) Convention on Biological Diversity, signed in Rio de Janeiro in 1992, requires member states “*as far as possible, and as appropriate, [to] prevent the introduction of, control or eradicate those alien species which threaten ecosystems, habitats or species*” [15]. One of the Convention’s aims, adopted in 2010, was that, by 2020, IAS and their pathways would have been identified and prioritized, priority species controlled or eradicated, and all the preventive measures to prevent their introduction and establishment would have been determined.

The UN targets were subsequently incorporated into the European Union (EU) biodiversity strategy [16], leading to the implementation of the EU Invasive Alien Species Regulation (Regulation (EU) No 1143/2014 of the European Parliament and of the Council of 22 October 2014 on the prevention and management of the introduction and spread of invasive alien species). The primary objective of this Regulation is to manage established IAS within the EU territories and to reduce, by 2030, the number of Red-Listed species threatened by IAS by 50% [17]. Despite these efforts, data collected by the European Alien Species Information Network (EASIN) catalogue indicate that more than 14,000 alien taxa are currently present within the European continent. Among these, 88 species are classified as IAS of EU concern and, for this reason, are strictly regulated. Of these 88 species, 47 are animals [17], including 14 invertebrates, 10 fishes, 1 amphibian, 2 reptiles, 5 birds, and 14 mammals.

In compliance with this Regulation, EU Member States are required to act on pathways of unintentional introduction, to take measures for the early detection and rapid eradication of these species (i.e., prevention), and to manage species that are already widely spread in their territory. Several examples demonstrate that, once implemented at the territorial level, this legislation has provided an essential framework for the prevention and management of IAS, supporting coordinated actions and pathway control across Member States [18]. In particular, regarding early detection, EASIN was developed to support the implementation of European policies on IAS by providing harmonized data on species distribution, introduction pathways, and impacts. It constitutes a key component of the EU’s Early Warning and Rapid Response (EWRR) system [19]. Furthermore, evidence from eradication efforts indicates that earlier detection of an invasive species substantially increases the feasibility and likelihood of successful eradication within the affected territory [20]. In practice, numerous projects funded under the EU LIFE Programme have been implemented. Among the most significant is the LIFE U-SAVEREDS project (LIFE13 BIO/IT/000204), which aimed to conserve the Apennine ecosystem and achieved the eradication of more than 70% of the gray squirrel (*Sciurus carolinensis*) population in the Italian region of Umbria, alongside a concurrent increase in the presence of the native European red squirrel (*Sciurus vulgaris*). A comparable initiative is the LIFE LAMPROPELTIS project (LIFE10 NAT/ES/000565), which resulted in the removal of approximately 5000 specimens of *Lampropeltis getula* from the island of Gran Canaria, Spain. Another notable example is the LIFE MIRDINEC project (LIFE09 NAT/SE/000344), which addressed biodiversity loss associated with the introduction of the raccoon dog (*Nyctereutes procyonoides*) in Scandinavia and led to the eradication of approximately 1700 animals over the course of the project.

Through these measures, the EU aims to protect biodiversity and ecosystem services from the negative effects caused by IAS, ensuring long-term ecological balance and sustainability.

While most alien species have limited ecological impact, a small subset of highly invasive taxa can cause substantial ecological threats [21]. Despite their limited number, IAS are now considered one of the main drivers of recent extinctions [22]. Recent studies have shown that the presence of a single highly invasive species in a newly introduced area can cause a decrease of up to 16.6% in species richness and biodiversity [23,24].

Besides their well-documented impact on biodiversity, IAS can profoundly alter socio-economics and ecological systems. One of the lesser known but increasingly evident effects involves the disruption of the ecological balances through both direct and indirect mechanisms. Invasive species may compete with native species for resources, such as food and shelter, subsequently altering the food web [25], leading to shifts in species composition, habitat structure and overall ecosystem functioning. These dynamics are not isolated phenomena: they unfold against a backdrop of accelerating environmental change. Climate change, land use modification, and habitat fragmentation are now recognized as primary facilitators of biological invasions, as they weaken the ecological resistance of recipient ecosystems, expand the climatic suitability of invaded areas, and reduce the buffering capacity of native biodiversity [26]. The compounding interaction between pre-existing environmental degradation and IAS establishment therefore produces ecological disruptions that are greater than either driver alone would generate [27]. Beyond ecological concerns, IAS impacts have relevance to human well-being and economic costs. Although the interplay between invasive species and local livelihoods is exceptionally complex and depends on social–ecological contexts, it is evident that the ecological alterations created by these species can have both detrimental and beneficial effects on human society and global welfare [28]. These socio-economic consequences are further mediated by environmental drivers: deforestation, agricultural expansion, and urbanization transform landscapes in ways that alter human exposure to disease hosts and vectors, simultaneously increasing vulnerability to IAS-driven ecological disruption, particularly in regions already facing resource pressure [29]. However, it is widely recognized that the most vulnerable populations, in particular, indigenous and local communities that are highly dependent on natural resources, are at greater risk of facing significant consequences for their well-being [11].

Among the most consequential, yet often overlooked, impacts of IAS is their potential to harbor and transmit pathogens, posing health threats to humans and native animals, as they can facilitate the emergence and spread of infectious diseases [30]. In addition, variability in host immune responses can significantly influence infection outcomes and transmission dynamics, with immune dysregulation being increasingly recognized as a key factor in susceptibility to emerging pathogens [31]. Once introduced and established in a new area, invasive hosts can act as reservoirs or amplifiers of novel pathogens, causing spillover events to native species, thereby increasing risks to wildlife populations and to overall biodiversity [32]. For instance, the raccoon (*Procyon lotor*) has introduced the nematode *Baylisascaris procyonis* into Europe. While raccoons are the definitive hosts, small mammals, birds, and, in rare cases, humans can act as paratenic hosts, where larval migration to vital organs may lead to a severe condition known as “*larva migrans syndrome*” [30]. Another well-known example is *Batrachocytrium dendrobatidis*, a fungal pathogen dramatically affecting amphibian populations worldwide. The presence of the fungus within African *Xenopus laevis* populations is well documented, and infection has also been demonstrated in introduced populations in the United States, presumably via the international live amphibian trade, representing a well-studied case of pathogen dissemination mediated by an invasive host [33]. Moreover, IAS can significantly disrupt the local epidemiological balances, both with direct and indirect mechanisms. Directly, non-native species can act as amplifiers of local pathogens when they are both susceptible to infection and capable of facilitating further transmission within their new environment. This is observed with certain bird species introduced into North America, such as the European starling (*Sturnus vulgaris*), which, even though not considered a natural reservoir for avian influenza, can play a role as local amplifiers or avian flu bridge hosts at the wildlife–poultry interface [34,35,36]. Indirectly, allochthonous species can alter the existing environmental conditions by engaging in competitive or trophic interactions with native species, thereby reshaping host–pathogen dynamics [24]. Consequently, many of the major invasive pathogen events have been associated with significant biological changes, often leading to the decline of native biodiversity [37].

Despite the recognized public health relevance of IAS interacting with zoonotic pathogens, their epidemiological role remains largely underestimated. Zoonotic surveillance in wildlife, and in IAS in particular, faces multiple challenges, including the asymptomatic or subclinical nature of many infections [38], the frequent lack of validated diagnostic tests for wild species [39], and the general absence of coordinated wildlife disease surveillance infrastructure and systematic sampling strategies [40,41]. Collectively, these limitations hinder early pathogen detection and accurate epidemiological assessment, resulting in significant underrepresentation of pathogen circulation in wildlife populations, leaving critical gaps in our understanding of the zoonotic risk posed by these species.

The available data were summarized to examine the potential impact of the pathogens included in key regulatory and public health frameworks, including the European Animal Health Law [42], the World Health Organization (WHO) “Pathogens Prioritization” (2024) [43], the “Fungal Priority Pathogens List” (2022) [44], and the “Bacterial Priority Pathogens List” (2024) [45] documents, and the EU “Zoonosis Directive” (99/2003/CE) [46]. This comparison with the results of the review provides an up-to-date overview of the European epidemiological context, highlighting zoonotic pathogens and their relevance from a One Health perspective. In the context of this study, priority pathogens are defined as those pathogens formally identified by EU and international public health and regulatory frameworks as posing significant threats to animal and human health. These frameworks prioritize pathogens according to their zoonotic potential, transmissibility, emergence, and disease severity.

However, despite growing evidence that IAS may act as reservoirs, amplifiers, or bridge hosts of pathogens, no comprehensive synthesis exists that is focused on IAS of Union concern and that integrates pathogen occurrence across terrestrial vertebrate taxa while prioritizing findings according to European and global public and animal health frameworks. To address this gap, the present study provides, to our knowledge, the first systematic review of high-priority pathogens reported in association with 22 terrestrial vertebrate IAS of EU concern and combines these data with a composite Host–Pathogen Influence Index (HPI-IAS) to identify spatial patterns of epidemiological pressure across EU Member States within a One Health framework.

## 2. Materials and Methods

### 2.1. Study Design and Species Selection

The present work was designed as a systematic review aimed at summarizing the current knowledge on pathogens affecting the IAS of Union concern present in Europe. The analysis focused on 22 terrestrial vertebrate species, among the 47 animal IAS of EU concern, including mammals, birds, reptiles and amphibians, while invertebrates and fish species were excluded. This selection was based on the well-known epidemiological role of terrestrial vertebrates as primary sources of emerging infectious disease. Previous studies have shown that most emerging infectious diseases are of zoonotic interest and originate predominantly from wildlife, particularly mammals and other vertebrate taxa [47,48,49]. Furthermore, these groups are more likely to interact directly with livestock, domestic animals and also humans in urban and peri-urban environments, thereby increasing the risk of pathogen transmission in the human–animal interface. Although invertebrates and fish are widely recognized as important components of disease transmission dynamics, they were excluded to maintain a focused and comparable framework centered on terrestrial vertebrate hosts directly involved in pathogen maintenance and spillover events.

The species of interest are: *Acridotheres tristis*, *Alopochen aegyptiacus*, *Axis axis*, *Callosciurus erythraeus*, *Callosciurus finlaysonii*, *Corvus splendens*, *Herpestes javanicus*, *Lampropeltis getula*, *Lithobates catesbeianus*, *Muntiacus reevesi*, *Myocastor coypus*, *Nasua nasua*, *Nyctereutes procyonoides*, *Ondatra zibethicus*, *Oxyura jamaicensis*, *Procyon lotor*, *Prycnotus cafer*, *Sciurus carolinensis*, *Sciurus niger*, *Eutamias sibiricus*, *Threskiornis aethiopicus*, *Trachemys scripta*.

### 2.2. Systematic Review of the Literature

A systematic review of the literature was carried out to investigate the pathogens affecting the IAS of Union concern. The literature search was conducted at a global scale, without geographic restrictions, in order to obtain the most comprehensive overview possible of pathogens reported in association with these species. The review was performed using PubMed (https://pubmed.ncbi.nlm.nih.gov/) and Scopus (https://www.scopus.com/pages/home#basic) (accessed on 6 April 2026) as primary search engines, applying the following terms: “*Scientific name of the species*” AND “*disease*” OR “*Scientific name of the species*” AND “*zoonosis*”; for example, “*Trachemys scripta*” AND “*disease*”. The term “*disease*” was used as a broad descriptor commonly adopted in the literature to identify host–pathogen studies.

Literature results were checked for duplicates using the Rayyan software available online (https://new.rayyan.ai/reviews) (accessed on 6 April 2026). The screening process was conducted independently by two reviewers (Luca Spadotto and Laura Cavicchioli) and consisted of two sequential stages. The first stage consisted of title and abstract screening, followed by a full-text assessment of the potentially relevant articles. All cases of uncertainty were resolved through discussion and consensus between reviewers. Interreviewer agreement was not formally quantified; however, the use of independent screening followed by consensus and discussion ensured consistency in the application of the eligibility criteria.

#### 2.2.1. Inclusion and Exclusion Criteria

Eligibility criteria were defined a priori in accordance with the Preferred Reporting Items for Systematic Reviews and Meta-Analyses (PRISMA) guidelines. Studies were considered eligible if they reported naturally acquired infections caused by bacteria, viruses, fungi, protozoa and endoparasites in at least one IAS. For the purposes of this review, natural infections that were defined as infection detected in animals under non-experimental conditions, without intentional or artificial exposure, without relevance to natural epidemiological dynamics were excluded.

Only peer-reviewed articles written in or containing an English abstract were included. Theses, conference proceedings and gray literature were excluded. No restrictions based on the year of publication were applied to ensure a comprehensive dataset. For each research article, information regarding host species, pathogen species, year, and geographic location of the study was collected.

#### 2.2.2. Quality Assessment and Risk of Bias

A formal assessment of study quality and risk of bias was not performed. This was a deliberate methodological choice, as the primary aim of this study was to provide a comprehensive overview of all pathogens reported in association with IAS, including evidence derived from single case reports. Given this objective, excluding or weighting studies based on methodological quality could have led to the omission of rare but potentially relevant host–pathogen associations. Therefore, all available evidence was considered, regardless of study design or sample size.

### 2.3. Data Integration and Prioritization of Pathogens of Public and Animal Health Significance in the European Context

Information on pathogens affecting IAS retrieved from the literature was cross-referenced with official documents from the EU and the WHO to identify the “High-Priority Pathogens”, defined as those of recognized significance to both public and animal health. For each pathogen, the inclusion criteria were considered met if included in the following documents: the European Animal Health Law (Reg. UE 2016/429) [42], subsequent Commission Implementing Regulation (EU) 2020/2002 [50], and Zoonoses Directive 2003/99/EC [46] annexes 1, list A and List B; the WHO’s 2024 “Pathogen Prioritization” document [43], focusing on their geographical distribution across the European, African, and Eastern Mediterranean regions and their Public Health Emergency of International Concern (PHEIC) status, and the WHO’s 2024 “Bacterial Priority Pathogens List” [45] and the WHO’s 2022 “Fungal Priority Pathogens List” [44].

The data extracted from these documents were harmonized to create a list of pathogens of animal and human health relevance. Pathogens described in one or more of these cited documents were classified as high-priority pathogens and subsequently used as references for the epidemiological risk assessment.

The following Table 1 summarizes all the priority pathogens described in the previous documents.

A second level of filtering was applied on a geographical basis, focusing exclusively on records reporting high-priority pathogens in IAS within the EU Member States. This approach allowed the analysis to concentrate on the relevance of IAS as potential pathogen hosts in Europe.

Finally, an operational epidemiological criterion was introduced to identify “Key IAS”. These were defined as the IAS for which documented scientific reports of pathogen occurrence are available within the EU.

### 2.4. Epidemiological Risk Analysis

For each EU Member State, information was compiled on the composition of its IAS assemblage using the EASIN database (https://easin.jrc.ec.europa.eu/spexplorer/ accessed on 23 May 2025). The dataset included the total number of IAS recorded per country (N _IAS, c_) and the subset classified as Key IAS based on predefined epidemiological and ecological relevance criteria (N _Key-IAS, c_).

#### 2.4.1. Assessment of Invasion Pressure and Spatial Expansion

To quantify ecological pressure and invasion dynamics, occurrence records were retrieved from the Global Biodiversity Information Facility (GBIF; https://www.gbif.org accessed 6 February 2026). Records were aggregated into standardized 10 × 10 km grid cells for three reference years: 2000, 2014, and 2026.

The year 2000 was selected to maximize occurrence data availability while ensuring temporal consistency [51] with previous European assessments. The year 2014 was included as a regulatory milestone corresponding to the entry into force of Regulation (EU) No. 1143/2014 on invasive alien species. The year 2026 represents the most recent distribution available at the time of analysis.

For each species (s) within a country (c), relative spatial expansion was quantified as the log-transformed ratio of occupied grid cells between the baseline (t0 = 2000 or 2014) and final year (t1 = 2026):

$$G_{c,s}=ln\left(\frac{W_{t1,c,s}+1}{W_{t0,c,s}+1}\right)$$

The addition of one grid cell avoids undefined values when a species was absent at baseline.

Country-level invasion growth was then calculated as the arithmetic mean across all IAS present in that country:

$$G_{c}=\frac{1}{S_{c}}\sum_{s=1}^{S_{c}}G_{c,s}$$

Because raw growth values may vary substantially across Member States due to differences in invasion history and spatial extent, relative expansion was standardized using min–max normalization:

$$S_{norm,c}=\frac{G_{c}-min(G)}{\max\left(G\right)-\min\left(G\right)}$$

where min(G) and max(G) were calculated across all Member States.

Biological pressure was calculated as:

$$P_{norm}=\frac{N_{IAS,c}}{{max}_{c}\left(N_{IAS,c}\right)}$$

which represents the normalized size of the IAS pool within each country. To integrate invasion magnitude with spatial growth dynamics, a composite invasion pressure term was defined as:

$$P^{*}=P_{norm}\times S_{norm}$$

This formulation captures both the cumulative size of the IAS assemblage and its current spatial expansion trajectory, thereby reflecting dynamic invasion pressure rather than static species richness alone.

#### 2.4.2. Epidemiologically Relevant IAS

The normalized proportion of Key IAS within each country was calculated as:

$$H_{norm,\;c}=\frac{N_{KeyIAS,c}}{{max}_{c}\left(N_{KeyIAS,c}\right)}$$

Absolute counts were preferred over proportional metrics to reflect epidemiological mass, ensuring that countries with larger numbers of high-risk IAS were not down-weighted solely because of overall IAS richness.

**Pathogen infectivity potential**

High-priority pathogens associated with IAS were identified through a systematic literature review and official epidemiological sources. For each pathogen (p), an epidemiological scoring system was applied based on four parameters:

- Host breadth (specialist, restricted multi-host, generalist),
- Transmission route complexity (complex/rare, moderate, direct/environmental),
- Life-cycle structure (obligate indirect, flexible indirect, direct),
- One Health impact level (low, high).

The first three parameters were scored ordinally (0–2), whereas the One Health parameter was coded as a binary variable (0–1). The total epidemiological score (ES_p_) for each pathogen was calculated as:

$${ES}_{p}=A_{p}+B_{p}+C_{p}+D_{p}$$

For each country, the potential epidemiological burden was computed as the sum of pathogen scores associated with IAS present within that country:

$${Lp}_{epiraw,c}=\sum_{p=1}^{k_{c}}{ES}_{p}$$

where kc denotes the number of IAS-associated pathogens recorded in a country (c). To ensure cross-country comparability, values were normalized as:

$${Lp}_{epinorm,c}=\frac{{Lp}_{epiraw,c}}{max\left({Lp}_{epiraw,c}\right)}$$

This component represents the relative epidemiological infectivity potential of IAS-associated pathogens.

**Observed pathogen load**

To complement theoretical infectivity potential, empirical pathogen evidence was incorporated. Pathogen occurrences documented within each country were extracted from peer-reviewed studies and official surveillance reports for Key IAS. To prevent artificial inflation due to multiple host–pathogen combinations, pathogen records were deduplicated at the country level.

Observed pathogen load was calculated as:

$${Lo}_{raw,\;c}=O_{Key,\;c}$$

where O_Key,c_ and represent observed pathogens in Key IAS.

Normalization was performed as:

$${Lo}_{norm,c}=\frac{{Lo}_{raw,\;c}}{max\left({Lo}_{raw,\;c}\right)}$$

This metric captures empirical pathogen detection rather than theoretical potential.

#### 2.4.3. Final Composite Index

All components were scaled between 0 and 1 to ensure comparability. The updated Host–Pathogen Influence Index for Invasive Alien Species (HPI-IAS) was defined as:

$${HPI-IAS}_{c}=0.30P_{c}^{*}+0.25H_{norm,\;c}+0.25{Lp}_{epinorm,c}+0.20{Lo}_{norm,c}$$

where:

- $P_{c}^{*}$ represents dynamic invasion pressure,
- $H_{norm,\;c}$ represents the proportion of epidemiologically relevant IAS,
- ${Lp}_{epinorm,c}$ represents pathogen infectivity potential,
- ${Lo}_{norm,c}$ represents observed pathogen evidence.

Weights were assigned to reflect the conceptual hierarchy underlying the index structure, with dynamic invasion pressure considered the primary driver (30%), followed by epidemiological relevance of IAS (25%), pathogen infectivity potential (25%), and empirical pathogen evidence (20%). The weighting scheme was designed to balance ecological dynamics with epidemiological risk while avoiding dominance by any single component.

Higher HPI-IAS values indicate stronger combined influence of IAS on pathogen introduction, establishment, and transmission potential within a country. For interpretative purposes, countries were classified into five categories (very low, low, moderate, high, very high) using quintile-based thresholds derived from the empirical distribution of HPI-IAS values.

The detailed construction of the HPI-IAS is provided in Tables S1–S5.

#### 2.4.4. Post Hoc Validation and Statistical Robustness Analysis

To evaluate the internal consistency and structural robustness of the HPI-IAS, a comprehensive post hoc validation framework was implemented following established composite indicator methodology [52]. Data processing and exploration were conducted in Python 3.10 (pandas, NumPy) [53,54,55].

First, index values were recalculated directly from their normalized components to verify computational accuracy and ensure full reproducibility of results, in line with recommended internal validation procedures for composite indicators [56]. This step confirmed the internal coherence between individual components and the final composite score.

To assess robustness to weighting assumptions, a Monte Carlo sensitivity analysis was performed [57,58]. Component weights were randomly perturbed within ±20–30% of their baseline values and subsequently renormalized to ensure that their sum remained equal to one, consistent with uncertainty propagation approaches commonly applied in composite index construction [52]. For each simulated weighting scheme, the HPI-IAS was recalculated and country rankings were compared to the original baseline ranking. Ranking stability was quantified using Spearman’s rank correlation coefficient and Kendall’s tau coefficient [59], which measure monotonic agreement between rankings and concordance of pairwise positions, respectively. In addition, mean absolute rank shifts were computed to quantify average positional variability across simulations, following established practices in robustness analysis of composite indicators [58].

Sensitivity to normalization methods was also examined. The index was recalculated using z-score standardization instead of min–max scaling, and the resulting rankings were compared with those obtained under the original normalization scheme. This approach follows recommendations that normalization choice may influence composite index outcomes and should therefore be explicitly tested [52,56]. High agreement between alternative normalization strategies was interpreted as evidence that results were not driven by scaling assumptions.

To further evaluate structural balance among components, leave-one-out analyses were conducted. Each component was sequentially removed, the remaining weights were renormalized, and country rankings were recalculated. This procedure allowed assessment of whether any single component exerted disproportionate influence on the composite score, a common diagnostic step in index robustness evaluation [42,58].

Collectively, these validation procedures provided a multi-layered assessment of ranking stability, component balance, and structural robustness.

## 3. Results

### 3.1. Systematic Review of the Literature

**Figure 1:**
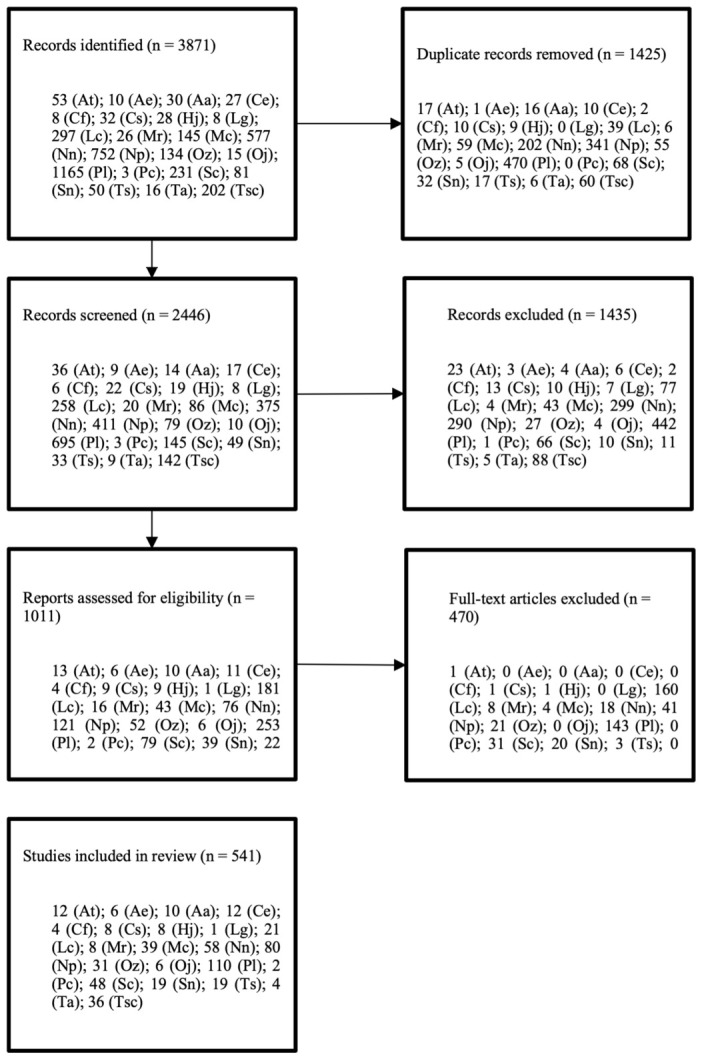
PRISMA flow describing the systematic review. At = *Acridotheres tristis*; Ae = *Alopochen aegyptiacus*; Aa = *Axis axis*; Ce = *Callosciurus erythraeus*; Cf = *Callosciurus finlaysonii*; Cs = *Corvus splendens*; Hj = *Herpestes javanicus*; Lg = *Lampropeltis getula*; Lc = *Lithobates catesbeianus*; Mr = *Muntiacus reevesi*; Mc = *Myocastor coypus*; Nn = *Nasua nasua*; Np = *Nyctereutes procyonoides*; Oz = *Ondatra zibethicus*; Oj = *Oxyura jamaicensis*; Pl = *Procyon lotor*; Pc = *Pycnonotus cafer*; Sc = *Sciurus carolinensis*; Sn = *Sciurus niger*; Ts = *Tamias sibiricus*; Ta = *Threskiornis aethiopicus*; Tsc = *Trachemys scripta*.

The literature search identified a total of 3871 records. After removing 1425 duplicates, 2446 articles were screened based on title and abstract, of which 1435 were excluded. The remaining 1011 articles were assessed for eligibility though full-text review. Of these, 470 studies were excluded due to multiple reasons, including experimental or artificial infections, irrelevance to target species, and lack of pathogen-related data. Finally, 541 studies, published between 1963 and 2023, were included in the review. The study selection process followed PRISMA guidelines and is summarized in Figure 1.

### 3.2. Pathogen Species in Invasive Alien Species (IAS)

Most of the studies identified were reports describing infectious pathogens in IAS, including some literature reviews on specific taxa. The following Table 2 represents a compiled pathogen record of all studied IAS of EU concern. Overall, 541 publications were analyzed, documenting a total of 472 pathogen taxa in the accounted IAS. These values are not directly comparable on a one-to-one basis, because a single study could report multiple pathogens, while the same pathogen could be reported in different studies and/or host species. The most investigated species were the raccoon (*Procyon lotor*), the raccoon dog (*Nyctereutes procyonoides*), the coati (*Nasua nasua*), the eastern gray squirrel (*Sciurus carolinensis*), and the nutria (*Myocastor coypus*), which together accounted for more than half of the total records. Bacteria and endoparasites were the most commonly reported pathogens, at 25.43% and 37.08% respectively. These were followed by viruses (18.64%) and protozoa (13.98%) while fungi were less frequently reported (4.87%).

**Figure 2:**
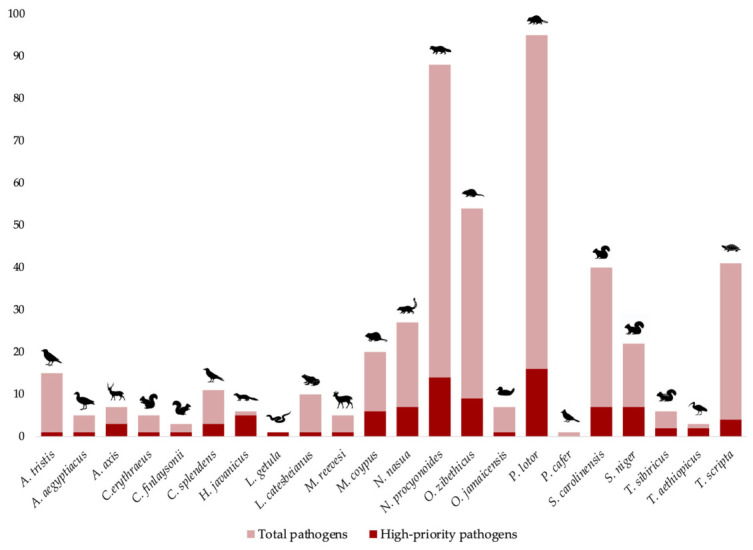
Graphic representation of the total pathogen richness per IAS and highlighting the high-priority pathogens.

This systematic review summarizes the pathogens reported on twenty-two IAS of EU interest. Based on the selected framework and directive documents, 112 prioritizing pathogens were identified (Table 1). Of these, 64 pathogens were reported in association with IAS in the reviewed literature and therefore considered as high-priority pathogens. These comprised bacteria, viruses, endoparasites, protozoa, and fungi (Figure 2). The reviewed literature reported the presence of pathogens in IAS that have been described as reservoirs, amplifiers, or vectors of many pathogens, some of which have zoonotic potential.

#### 3.2.1. High-Priority Bacteria Species in IAS

Among the bacterial pathogens of primary concern found in IAS, more than ten species of bacteria have been identified, notably, *Borrelia* spp., *Brucella* spp., *Campylobacter* spp., *Francisella* spp., *Klebsiella* spp., *Leptospira* spp., *Salmonella* spp., and *Yersinia* spp. (Table 3). Many of these are zoonotic pathogens linked to disease both in humans and in animals. Eight species of *Borrelia* have been identified in four EU countries, spanning between southern and western-central Europe, Italy, France, Denmark, and Poland. All records originated from mammalian hosts, including the raccoon dog (*Nyctereutes procyonoides*), the raccoon, the eastern gray squirrel (*Sciurus carolinensis*), and the Siberian chipmunk (*Tamias sibiricus*). The occurrence of *Borrelia* in invasive raccoons and raccoon dogs has been extensively studied especially in Poland, with focus on the relation with other native wildlife species. Two independent Polish studies returned remarkably similar results, each reporting a positivity rate of 62.5% among the tested animals [60,61]. Similarly, in France, the epidemiological role of the Siberian chipmunk as a potential reservoir has been the subject of multiple studies, which demonstrated a significant presence of the pathogen. Findings indicate that up to 47% of the examined specimens tested positive [62,63,64].

Another widely reported pathogen was *Campylobacter*. It has been found in five different countries in North America and Asia, with occurrences noted in the Asian mongoose (*Herpestes javanicus*), the raccoon dog, the raccoon, the muskrat (*Ondatra zibethicus*), and in the Indian house crow (*Corvus splendens*). Among these, the most notable findings originate from Japan, where 75% of the examined raccoon dogs tested positive for *Campylobacter upsaliensis* [65]. Comparable infection rates were observed in the United States and Canada, where two independent studies have yielded comparable results in two different species, the raccoon and the muskrat, demonstrating that *Campylobacter* infection is a common finding in wildlife in this region. Indeed, in Canada, 46% of raccoons tested positive [66], while in the United States, the incidence of positivity of the sampled muskrats was around 47% [67].

*Francisella tularensis*, the causative agent of tularemia, has been identified in several IAS, notably among rodents. Indeed, muskrats, eastern gray squirrels, and fox squirrels (*Sciurus niger*) proved to have a role in tularemia epidemiology [68,69,70] in North America, with a positivity range of 51.7% in gray squirrels and 33% in fox squirrels. In addition to rodents, raccoons and raccoon dogs have also shown positivity for *F. tularensis* [71,72,73]. Within the EU, *Francisella* infections have been primarily observed in the Baltic area, particularly in the raccoon dog [71,74,75,76].

Moreover, all IAS carnivores appear susceptible to *Leptospira interrogans*, and ongoing research is evaluating their role as reservoirs [77,78,79,80]. *Leptospira* has been detected in a range of mammalian IAS, including the Asian mongoose, the coati (*Nasua nasua*), the raccoon dog and the raccoon, the nutria (*Myocastor coypus*), the eastern gray squirrel, the fox squirrel, and also in the red-eared slider (*Trachemys scripta*). Within Europe, *Leptospira* spp. has been reported in six countries, mainly in Western Europe. Research on the incidence of *Leptospira* has produced alarming results, and several studies carried out on nutrias between France and the Netherlands have shown a remarkably high positivity rate according to the different areas examined, ranging between 16.5 and 81% [81,82,83,84]. Outside of Europe, several studies conducted in the United States confirmed high *Leptospira* positivity rates in wildlife, with prevalence in raccoons ranging from 11% to 48% [74,85,86,87] and a consistently high rate of approximately 42–45% observed in fox squirrels [88,89].

Lastly, *Salmonella* spp. has been detected in a diverse range of IAS hosts, mainly mammals such as the Asian mongoose, the raccoon, and the eastern gray squirrel. It has also been detected in birds, most notably the common myna (*Acridotheres tristis*), and in reptiles, including the red-eared slider. The epidemiological significance of *Salmonella* in IAS has been intensively studied, particularly in raccoons in North America, where reported positivity rates range from 15% to 63% [90,91,92,93]. Further research focusing on the invasive turtle *Trachemys scripta* in both in China and the United States revealed consistent positivity rates of approximately 38–39% [94,95], reinforcing the recognized role of reptiles in harboring and potentially disseminating the pathogen [96]. According to the literature, *T. scripta* is predominantly affected by infections with *Salmonella enterica*, involving different serovars such as Paratyphi B, Typhimurium, Thompson, and Muenchen [97,98,99]. Additionally, a case of infection with the highly pathogenic *S. enterica* serovar Pomona was described in China in 2014 [100]. In avian IAS, *Salmonella* has been recorded limitedly in common mynas and house crows [101,102], which as wild birds can serve as reservoirs or vectors for *Salmonella* transmission [103].

**Table 3:** High-priority bacteria species in IAS.

Bacteria	Document	IAS Involved	Pathogen Species	Location EU	Location EXTRA EU
*Borrelia* spp.	Directive Zoonoses 2003/99/EC	**Mammals**: *Procyon lotor*, *Nyctereutes procyonoides*, *Sciurus carolinensis*, *Sciurus niger*, *Tamias sibiricus*	*B.* spp., *B. afzelli*, *B. burgdorferi*, *B. garinii*, *B. lonestarii*, *B. lusitaniae*, *B. miyatomotoi*, *B. theileri*, *B. turicatae*, *B. valaisiana*	**France** (Vourc’h et al. (2007) [62], Marsot et al. (2011) [64], Marsot et al. (2013) [104], Marchant et al. (2017) [63]), **Denmark** (Kjær et al. (2021) [105]), **Italy** (Cruciani et al. (2022) [106]), **Poland** (Wodecka et al. (2016) [60], Myśliwy et al. (2022) [61]).	**Republic of Korea** (Kang et al. (2018) [107], Myśliwy et al. (2022) [61]), **UK** (Millins et al. (2015) [108]), **USA** (Salkeld et al. (2008) [109], Roy et al. (2017) [110]).
*Brucella abortus*	Animal Health Law Reg. UE 2016/429	**Mammals**: *Nasua nasua*	*B. abortus*		**Brazil** (Oliveira-Filho et al. (2012) [111]).
*Brucella* spp.	Directive Zoonoses 2003/99/EC	**Mammals**: *Nasua nasua*	*B. abortus*, *B. canis*		**Brazil** (Oliveira-Filho et al. (2012) [111]).
*Campylobacter* spp.	Directive Zoonoses 2003/99/EC	**Mammals**: *Herpestes javanicus*, *Nyctereutes procyonoides*, *Procyon lotor*, *Ondatra zibethicus*. **Birds**: *Corvus splendes*	*C.* spp., *C. jejuni*, *C. coli*		**Barbados** (Rhynd et al. 2014) [77]), **Canada** (Mutschall et al. (2020) [66]), **Japan** (Lee et al. (2011) [112], Takako et al. (2021) [65]), **Malaysia** (Ganapathy et al. 2007) [102]), **USA** (Pacha et al. (1985) [67], Rainwater et al. (2017) [90]).
*Chlamydia psittaci*	Directive Zoonoses 2003/99/EC	**Mammals**: *Myocastor coypus*	*C. psittaci*		**Argentina** (Martino et al. (2014) [113]).
*Clostridium botulinum*, *C. butyricum*, *C. baratii*	Directive Zoonoses 2003/99/EC	**Reptiles**: *Trachemys scripta*	*C. butyricum* type E, *C. butyricum* BoNT/E	**Ireland** (Shelley et al. (2015) [114]).	
*Francisella* spp.	Directive Zoonoses 2003/99/EC	**Mammals**: *Nyctereutes procyonoides*, *Procyon lotor*, *Ondatra zibethicus*, *Sciurus carolinensis*, *Sciurus niger*	*F. tularensis*	**Germany** (Kuehn et al. (2013) [76]), **Sweden** (Hestvik et al. (2019) [71]).	**Canada** (Ganoe et al. (2020) [68]), **USA** (Bischof and Rogers (2005) [75], Duncan et al. (2012) [74], Nelson et al. (2014) [69], Vincent et al. (2020) [115], Ganoe et al. (2020) [68], Jahan et al. (2021) [70]).
*Klebsiella pneumoniae*	WHO Pathogens Prioritization	**Mammals**: *Ondatra zibethicus*. **Birds**: *Corvus splendens*	*K. pneumoniae*		**Bangladesh** (Hasan et al. (2015) [116]), **USA** (Grear et al. (2019) [117]).
*Leptospira* spp.	Directive Zoonoses 2003/99/EC	**Mammals**: *Herpestes javanicus*, *Nasua nasua*, *Nyctereutes procyonoides*, *Procyon lotor*, *Myocastor coypus*, *Ondatra zibethicus*, *Sciurus carolinensis*, *Sciurus niger.***Reptiles**: *Trachemys scripta*	*L. interrogans*	**Belgium** (Mori et al. (2015) [118]), **France** (Michel et al. (2001) [81], Vein et al. (2014) [83]), **Germany** (Hurd et al. (2017) [119], Reinhardt et al. (2023) [80]), **Italy** (Bollo et al. (2003) [120], Bonacina et al. (2021) [121]), **The Netherlands** (Kik et al. (2006) [82]), **Slovenia** (Žele-Vengušt et al. (2021) [122]).	**Brazil** (Lilenbaum et al. (2002) [123], Minervino et al. (2018) [124], Fornazari et al. (2018) [78], Cutolo et al. (2020) [125]) **Canada** (Jardine et al. (2011) [126]), **Costa Rica** (Baldi et al. (2019) [127]), **Japan** (Yamashita et al. (2021) [79]), **Saint Kitts and Nevis** (Shiokawa et al. (2019) [128]), **USA** (Mitchell et al. (1999) [87], Hamir et al. (2001) [129], Richardson and Gauthier (2003) [130]; Bischof and Rogers (2005) [75], Raizman et al. (2009) [86], Duncan et al. (2012) [74], Dirsmith et al. (2013) [88], Tan et al. (2014) [85], Pedersen et al. (2018) [84], Straub et al. (2020) [131], Anderson et al. (2022) [89], Helman et al. (2023) [132]).
*Listeria monocytogenes*	Directive Zoonoses 2003/99/EC	**Mammals**: *Nyctereutes procyonoides*, *Procyon lotor.*	*L. monocytogenes*		**Japan** (Aoyagi et al. (2000) [133]), **USA** (Hamir et al. (2000) [134]).
*Mycobacterium bovis* complex	Directive Zoonoses 2003/99/EC Animal Health Law Reg. UE 2016/429	**Mammals**: *Muntiacus reevesi*, *Axis axis*, *nasua nasua*, *Procyon lotor*. **Amphibians**: *Lithobates catesbeianus*	*M. bovis*, *M. orygis*	**Poland** (Bruczyńska et al. (2022) [135])	**Brazil** (Murakami et al. (2012) [136], Albertti et al. (2015) [137], **India** (Refaya et al. (2022) [138]), **UK** (Ward et al. (2009) [139]), **USA** (Bruning-Fann et al. (2001) [140], Palmer et al. (2002) [141], Witmer et al. (2010) [142]).
*Salmonella* spp.	Directive Zoonoses 2003/99/EC	**Mammals**: *Procyon lotor*, *Herpestes javanicus*, *Procyon lotor*, *Sciurus carolinensis*. **Birds**: *Acridotheres tristis*. **Reptiles**: *Trachemys scripta*	*S.* spp., *S. enterica*	**Spain** (Marin et al. (2013) [98]).	**Australia** (Chang et al. (2021) [101]), **Barbados** (Rhynd et al. (2014) [77]), **Canada** (Bondo et al. (2016) [91], Bondo et al. (2019) [143], Vogt et al. (2021) [144]), **China** (Gong et al. (2014) [94]), **Japan** (Nagano et al. (2006) [97], Lee et al. (2011) [112]), **USA** (Jijón et al. (2007) [145], Gaertner et al. (2008) [93], Very et al. (2016) [92], Rainwater et al. (2017) [90], Jahan et al. (2021) [70], Vogt et al. (2022) [146]).
*Salmonella enterica* subsp. enterica serotype Enteritidis, *S*. Typhimurium, *S*. Newport, *S*. Heidelberg, *S*. Javiana	WHO Pathogens Prioritization	**Mammals**: *Herpestes javanicus*	*S. enterica* subsp. Enterica serotype Javiana		**Barbados** (Rhynd et al. (2014) [77]).
*Yersinia pestis*	WHO Pathogens Prioritization	**Mammals**: *Sciurus niger*	*Y. pestis*		**USA** (Malberg et al. (2012) [147]).
*Yersinia* spp.	Directive Zoonoses 2003/99/EC	**Mammals**: *Axis axis*, *Nyctereutes procyonoides*, *Procyon lotor.*	*Y. pseudotubercolosis*		**Australia** (Jerrett et al. (1990) [148]), **Japan** (Fukushima and Gomyoda (1991) [149]).

#### 3.2.2. High-Priority Virus Species in IAS

In addition to bacterial threats, nine high-priority viral agents have been identified in alien species, including *avian influenza virus*, *foot-and-mouth disease virus*, *rabies virus*, *West Nile virus*, *Japanese encephalitis virus*, and *hantavirus* (Table 4).

Among these, avian influenza (AI) has mainly been found in a variety of avian IAS, notably the common myna, Egyptian goose (*Alopochen aegyptiaca*), the Indian house crow, the ruddy duck (*Oxyura jamaicensis*), and the African sacred ibis (*Threskiornis aethiopticus*). However, its presence has been demonstrated not only in avian hosts but also in mammals, where it has been detected in the raccoon dog and the raccoon, confirming that the virus can spill over outside the original hosts. These high-pathogenicity (HPAI) strains have been investigated in several studies conducted in different regions of the world, including South and East Asia, North America, and Africa [150,151,152,153,154].

Rabies, a viral infection specific to mammals caused by *Lyssavirus rabies*, has been identified in several invasive mammals, including the coati, the raccoon dog, the raccoon, and the muskrat [83,155,156,157,158]. Studies conducted in North America and in Asia indicate that raccoons, in their native range, are considered the most commonly reported rabid wildlife species [159], while in Asia, raccoon dogs are a well-documented host for the virus [160]. This disease remains a significant concern in Europe, given that rabies surveillance programs have confirmed infections in the raccoon dog in the Baltic region, where this IAS is used as a model animal for virus detection with both active and passive surveillance [157,161,162].

Also, *West Nile virus* (WNV), an arthropod-borne pathogen, has been reported in multiple IAS, the raccoon, the eastern gray squirrel, and the fox squirrel. The virus is endemic across several EU countries, particularly in Central and Eastern Europe and also in the Mediterranean basin [163]. Lastly, one study in northern Italy reported a seroprevalence of 0.6% in the eastern gray squirrel [164].

**Table 4:** High-priority virus species in IAS.

Virus	Document	IAS Involved	Pathogen Species	Location EU	Location EXTRA EU
*Alphainfluenzavirus influenzae*	Directive Zoonoses 2003/99/EC Animal Health Law Reg. UE 2016/429 WHO Pathogens Prioritization	**Mammals**: *Nyctereutes procyonoides*, *Procyon lotor* **Birds**: *Alopochen aegyptiacus*, *Corvus splendens*, *Oxyura jamaicensis*, *Threskiornis aethiopicus*	Avian influenza virus		**Bangladesh** (Hassan et al. (2017) [150]), **China** (Qi et al. (2009) [154]), **India** (Verma et al. (2023) [151]), **Japan** (Yamaguchi et al. (2018) [165]), **Oman** (Body et al. (2015) [166]), **South Africa** (Thompson et al. (2008) [167], Burger et al. (2012) [168], Abolnik et al. (2016) [153]), **USA** (Hall et al. (2008) [169], Spackman et al. (2017) [152]).
*Apthovirus vesciculae*	Animal Health Law Reg. UE 2016/429	**Mammals**: *Axis axis*	Foot and mouth virus		**India** (Rout et al. (2017) [170]).
*Betacoronavirus pandemicum*	WHO Pathogens Prioritization	**Mammals**: *Nasua nasua*, *Nyctereutes procyonoides*	SARS-CoV-2		**Brazil** (Stoffella-Dutra et al. (2023) [171]), **India** (Sharun et al. (2021) [172]).
*Caliciviridae*	Directive Zoonoses 2003/99/EC	**Birds**: *Acridotheres tristis*	Calicivirus		**Australia** (Chang et al. (2021) [101]).
*Lyssavirus rabies*	Directive Zoonoses 2003/99/EC	**Mammals**: *Herpestes javanicus*, *Nasua nasua*, *Nyctereutes procyonoides*, *Ondatra zibethicus*, *Procyon lotor*	Rabies virus	**Lithuania** (Zienius et al. (2011) [157]), **Estonia** (Kulonen and Boldina (1993) [162]), **Finland** (Holmala and Kauhana (2009) [161]).	**Brazil** (Araujo et al. (2014) [173]), **Canada** (Ganoe et al. (2020) [68]), **China** (Wang et al. (2014) [156]), **USA** (Puskas et al. (2010) [158], Ganoe et al. (2020) [68]).
*Orthoflavivirus japonicum*	Animal Health Law Reg. UE 2016/429	**Mammals**: *Nyctereutes procyonoides*, *Procyon lotor*	Japanese encephalitis virus		**Japan** (Ohno et al. (2009) [174])
*Orthoflavivirus nilense*	Animal Health Law Reg. UE 2016/429	**Mammals**: *Procyon lotor*, *Sciurus carolinensis*, *Sciurus niger*	West Nile virus	**Italy** (Romeo et al. (2018) [164]).	**USA** (Kiupel et al. (2003) [175], Dietrich et al. (2005) [176], Heinz-Taheny et al. (2004) [177], Padgett et al. (2007) [178]).
*Orthohantavirus hantanense*	WHO Pathogens Prioritization	**Mammals**: *Myocastor coypsu*	Hantavirus		**Brazil** (Lemos et al. (2004) [179]).
*Varicellovirus suidalpha 1*	Animal Health Law Reg. UE 2016/429	**Mammals**: *Procyon lotor*	Aujesky virus		**USA** (Kirkpatrick et al. (1980) [180]).

#### 3.2.3. High-Priority Endoparasites in IAS

The two primary endoparasite species affecting IAS are *Echinococcus multilocularis* and *Trichinella* (Table 5), both of which have proven zoonotic potential. Based on different studies, nutrias and muskrats can act as intermediate hosts for the pathogens [181,182,183,184,185,186,187]. Within Europe, the parasites have been documented in both Baltic countries, Estonia, Latvia, Lithuania, and Poland, and in Western EU nations, including France, Belgium, and the Netherlands. Findings in raccoon dogs showed a similar incidence in three neighboring countries (Poland, Lithuania, and Latvia), where the positivity rate among the studied animals was around 8–10% [188,189,190]. Further studies support these results, as a Belgian survey reported *E. multilocularis* in 22% of the tested muskrats [188]. In comparison, a Russian study in the Selenga River delta identified characteristic cystic parasitic lesions in 5% of the examined muskrats [185]. The presence of *E. multilocularis* in muskrats in Russia and Switzerland confirms that rodent reservoirs of the pathogen exist in proximity to the EU.

*Trichinella*, by contrast, has been identified in only two IAS, the raccoon dog and the raccoon, across regions including Japan, Russia, the United States, and the Baltic countries. The highest prevalence was recorded in Europe, particularly in the raccoon dog, and studies conducted in Poland, Finland, Estonia, and Lithuania revealed positivity rates ranging from 28 to 57% [190,191,192,193]. Similarly, a Russian study found that 42% of the tested raccoon dogs were infected with the parasite [194]. In the United States, research on raccoons in Wisconsin also reported significant results, with approximately 19% of the animals testing positive for the pathogen [195].

**Table 5:** High-priority endoparasite species in IAS.

Parasites	Document	IAS Involved	Pathogen Species	Location EU	Location EXTRA EU
*Echinococcus multilocularis*	Directive Zoonoses 2003/99/EC Animal Health Law Reg. UE 2016/429 Directive	**Mammals**: *Nyctereutes procyonoides*, *Myocastor coypus*, *Ondatra zibethicus*	*E. multilocularis*	**Belgium** (Mathy et al. (2009) [186]), **Estonia** (Süld et al. (2014) [196], Laurimaa et al. (2015) [197]), **France** (Boussinesq et al. (1986) [198], Umhang et al. (2013) [181], Umhang et al. (2013) [182], Umhang et al. (2016) [183]), **Latvia** (Bagrade et al. (2016) [189]), **Lithuania** (Mažeika et al. (2009) [187], Bružinskaitė-Schmidhalter et al. (2012) [190]), **The Netherlands** (Maas et al. (2016) [199]), **Poland** (Pilarczyk et al. (2022) [188])	**Russia** (Masur and Fomina (2012) [185]), **Switzerland** (Thiele et al. (2023) [184])
*Trichinella* spp.	Directive Zoonoses 2003/99/EC	**Mammals**: *Nyctereutes procyonoides*, *Procyon lotor*,	*T*. spp., *T. britovi*, *T. murrelli*, *T. nativa*, *T. pseudospiralis*, *T. spiralis*	**Estonia** (Kärssin et al. (2017) [193]), **Finland** (Airas et al. (2010) [191]), **Germany** (Langner et al. (2022) [200]), **Lithuania** (Bružinskaitė-Schmidhalter et al. (2012) [190]), **Netherlands** (Maas et al. (2016) [199]), **Poland** (Cybulska et al. (2019) [192])	**Japan** (Kobayashi et al. (2007) [201]), **Russia** (Seryodkin et al. (2020) [194]), **USA** (Hill et al. (2008) [195])

#### 3.2.4. High-Priority Protozoa Species in IAS

Protozoan pathogens of primary concern include *Cryptosporidium*, *Sarcocystis*, *Toxoplasma*, and *Trypanosoma* (Table 6). Among these, *Cryptosporidium* has primarily been identified in North America and Asia across eight different IAS. Six of these are mammals, including the raccoon dog, raccoon, muskrat, eastern gray squirrel, fox squirrel, and Pallas’s squirrel. The remaining two species include a bird, the common myna, and a reptile, the eastern king snake (*Lampropeltis getula*). Concerning *Cryptosporidium*, the most notable studies focused on three species in different geographical settings; from a Chinese study performed on the raccoon dog, the incidence of positivity reached 37% for *C. canis* [202]; American investigations were performed on the fox squirrel and eastern gray squirrel, yielding results of positivity for the pathogen between 28 and 40% for the two species [203]. Moreover, the third most significant study concerns the eastern king snake; a survey carried out in Thailand gave positive results for *C. serpentis* in about 22% of the specimens tested [204]. Lastly, from the study by Rzezutka et al. emerges the infection of a single asymptomatic pond slider with *Cryptosporidium parvum* [205].

Among IAS, *Toxoplasma gondii* has primarily been identified in Central European countries, including Poland, Germany, and the Czechia, as well as in Italy. It has been identified in three non-native mammalian species: the raccoon dog, the nutria, and the muskrat. The prevalence rates of *Toxoplasma* among these species are relatively high, ranging from 24% to 59% [105,122,206,207]. Outside Europe, the involvement of IAS as intermediate hosts has been reported in multiple studies, notably in the raccoon dog, raccoon, muskrat, and two sciurids, the eastern gray squirrel and the fox squirrel. Additionally, the common myna, a bird IAS, has also been reported in a study from the Middle East. From these studies, the most important findings come from studies conducted in the United States, in raccoons, where prevalence rates ranged from 49% to 65% [87,90,208]. Fortunately, no cases of trypanosomiasis have been reported in IAS at the EU level to date. The pathogen has been found in Southeast Asia in chital [209], in Brazil in several studies involving the coati [210,211,212,213], and in the United States, where it has been identified in raccoons [214,215,216,217].

**Table 6:** High-priority protozoa species in IAS.

Protozoa	Document	IAS Involved	Pathogen Species	Location EU	Location EXTRA EU
*Cryptosporidium* spp.	Directive Zoonoses 2003/99/EC	**Mammals**: *Callosciurus erythraeus*, *Myocastor coypus*, *Nyctereutes procyonoides*, *Ondatra zibethicus*, *Procyon lotor*, *Sciurus carolinensis*, *Sciuris niger*, *Tamias sibiricus.***Birds**: *Acridotheres tristis* **Reptiles**: *Lampropeltis getula*, *Trachemys scripta*	*C*. spp., *C. canis*, *C. myocastoris*, *C. muris*, *C. parvum*, *C. serpentis*, *C. ubiquitum*, *C. wrairi*	**Czechia** (Hůrková et al. (2003) [218], Ježková et al. (2021) [219]), **Italy** (Lombardo et al. (2023) [220]), **Germany** (Rentería-Solís et al. (2020) [221]), **Poland** (Pavlásek and Kozakiewicz (1991) [222], Rzeżutka et al. (2020) [205]), **Slovakia** (Ježková et al. (2021) [219])	**Canada** (Ganoe et al. (2020) [68]), **China** (Qi et al. (2011) [223], Chai et al. (2019) [224], Wang et al. (2022) [202]), **Iran** (Mohammad Rahimi et al. (2022) [225]), **Japan** (Masuda et al. (2021) [226]), **Thailand** (Yimming et al. (2016) [204]), **USA** (Perz and Le Blancq (2001) [227], Stenger et al. (2015) [203], Stenger et al. (2018) [228], Ganoe et al. (2020) [68], Davis et al. (2022) [229])
*Sarcocystis* spp.	Directive Zoonoses 2003/99/EC	**Mammals**: *Ondatra zibethicus*, *Procyon lotor*	*S. jaypeedubey*, *S. kirkpatricki*, *S. neurona*, *S. sebeki*	**Germany** (Stolte et al. (1996) [230])	**Canada** (Ganoe et al. (2020) [68]), **USA** (Hamir et al. (1999) [231], Dubey et al. (2001) [232], Hamir and Dubey (2001) [233], Stanek et al. (2002) [234], Dryburgh et al. (2015) [235], Ganoe et al. (2020) [68], Gupta et al. (2023) [236])
*Toxoplasma* spp.	Directive Zoonoses 2003/99/EC	**Mammals**: *Myocastor coypus*, *Nyctereutes procyonoides*, *Ondatra zibethicus*, *Sciurus carolinensis*, *Sciurus niger*	*T. gondii*	**Czechia** (Hejlíček et al. (1997) [206]), **Denmark** (Kjær et al. (2021) [105]), **Germany** (Beltrán-Beck et al. (2012) [73]), **Italy** (Bollo et al. (2003) [120], Nardoni et al. (2011) [207])	**Canada** (Ganoe et al. (2020) [68]), **China** (Zheng et al. (2017) [237], Qin et al. (2020) [238]), **Israel** (Salant et al. (2013) [239]), **USA** (Mitchell et al. (1999) [87], Dubey et al. (2006) [240], Dubey et al. (2007) [241], Dubey et al. (2008) [242], Rainwater et al. (2017) [90], Kumar et al. (2019) [243], Ganoe et al. (2020) [68])
*Trypanosoma envasi*	Animal Health Law Reg. UE 2016/429	**Mammals**: *Nasua nasua*	*T. envasii*		**Brazil** (Alves et al. (2011) [213], Alves et al. (2016) [212], Martins Santos et al. (2018) [211], de Oliveira Porfirio et al. (2018) [210])

#### 3.2.5. High-Priority Fungal Species in IAS

Lastly, five high-priority fungal pathogens were detected in the literature affecting IAS (Table 7). Among these, *Candida albicans* and *Cryptococcus neoformans* are listed in the critical group of concern by the WHO, as they are opportunistic pathogens that cause infections in immunocompromised individuals, leading to severe conditions and death; although, at present, these two pathogens have not manifested high antifungal resistance [44]. In Europe, specifically in studies conducted in Spain and Italy, four species of pathogenic fungi have been identified in association with IAS. In Italy, two independent studies examined non-native squirrel species to assess their potential impact on human and animal health. In central Italy, *Candida albicans* has been detected in the eastern gray squirrel [106], while in southern Italy, *Cryptococcus neoformans* has been isolated from the variable squirrel (*Callosciurus finlaysonii*) [244]. Meanwhile, research in Spain focused on the invasive *Trachemys scripta*, where two fungal species belonging to the genus *Fusarium*, *F. keratoplasticum* and *F. falciforme*, were identified [245]. Beyond Europe, fungal pathogens were also sporadically reported. In Brazil, a study on coatis revealed high prevalence of *Histoplasma* infection, where about 86% of the animals examined tested positive, a result considered consistent with the high human rate of infection in the country [246]. While in Canada, *Cryptococcus gattii* was detected in nasal swabs of two eastern gray squirrels [247].

### 3.3. Spatial Distribution and Epidemiological Risk of High-Priority Pathogens in Europe and in the World

**Figure 3:**
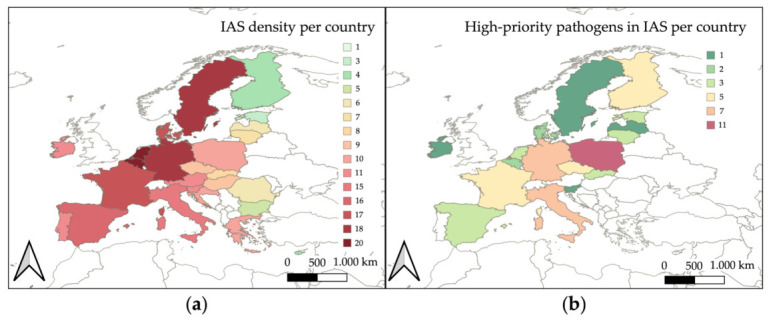
(**a**) IAS density in European countries according to the EASIN database, (**b**) High-priority pathogens associated with IAS per country.

Based on the EASIN database of the distribution of IAS within the EU [248], the highest IAS densities are recorded in Belgium and the Netherlands (20), Germany and Sweden (18), France and Denmark (17), Spain (16), and Italy (15), as illustrated in Figure 3a. These data were then compared with the distribution of primary pathogens identified in the present review (Figure 3b). This comparison revealed that the countries reporting the highest occurrence of these high-priority pathogens in IAS were Poland (11), Italy and Germany (7), France, Finland and Czechia (5), Lithuania, the Netherlands, Estonia, Slovakia, and Spain (3), Belgium and Denmark (2) and Latvia, Ireland, Sweden and Slovenia (1), whereas in Austria, Bulgaria, Croatia, Cyprus, Greece, Hungary, Luxembourg, Malta, Portugal, and Romania, the presence of high-priority pathogens in alien species has not been documented.

**Figure 4:**
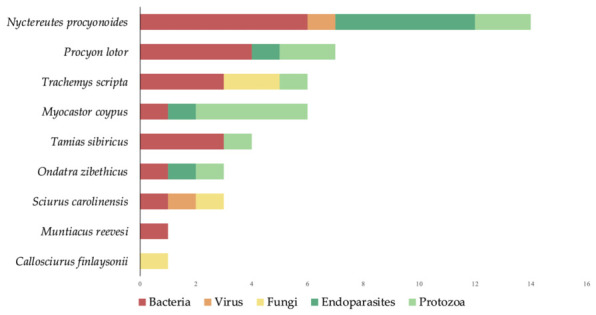
Visualization of high-priority pathogens reported in invasive alien species in Europe.

By cross-referencing the geographical information available in the reviewed literature, with the occurrence of IAS in the EU, nine alien species were identified as Key IAS associated with high-priority pathogens in Europe: *Callosciurus finlaysonii*, *Myocastor coypus*, *Muntiacus reevesi*, *Nyctereutes procyonoides*, *Ondatra zibethicus*, *Procyon lotor*, *Sciurus carolinensis*, *Tamias sibiricus*, *and Trachemys scripta*, as detailed in Table 8 and illustrated in Figure 4. These species were associated with multiple pathogen groups, including bacteria, viruses, fungi, endoparasites, and protozoa.

Finally, to provide a comprehensive overview of the epidemiological risk associated with Key IAS and high-priority pathogens across the EU, a composite risk score, the HPI-IAS, was calculated for each Member State. This scoring framework enabled the classification of the countries in distinct risk categories, where the epidemiological surveillance efforts should be activated, enhanced and integrated into structured monitoring programs.

**Figure 5:**
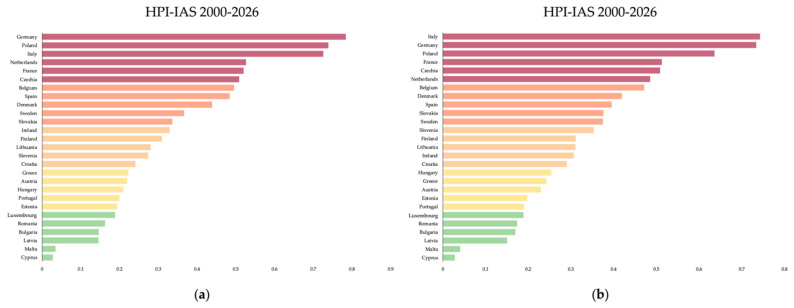
Graphical representation of the epidemiological risk calculated for all European Union member states, ranked from highest to lowest risk. (**a**) Calculated in the period between 2000–2026. (**b**) Calculated in the period 2014–2026.

Based on the analysis covering the period 2000–2016, Member States were grouped into the following categories: very high-risk (Germany, Poland, Italy, the Netherlands, France, and Czechia), high-risk (Belgium, Spain, Denmark, Sweden, and Slovakia), moderate-risk (Ireland, Finland, Lithuania, Slovenia, and Croatia), low-risk (Greece, Austria, Hungary, Portugal, and Estonia), and very low-risk (Luxembourg, Romania, Bulgaria, Latvia, Malta, and Cyprus) (Figure 5a; Table S1).

When the analysis was restricted to the period 2014–2026, a slightly different risk distribution emerged: very high-risk (Italy, Germany, Poland, France, Czechia, and the Netherlands), high-risk (Belgium, Denmark, Spain, Slovakia, and Sweden), moderate risk (Slovenia, Finland, Lithuania, Ireland, and Croatia), low-risk (Hungary, Greece, Austria, Estonia, and Portugal), and very low-risk (Luxembourg, Romania, Bulgaria, Latvia, Malta, and Cyprus) (Figure 5b; Table S2).

**Figure 6:**
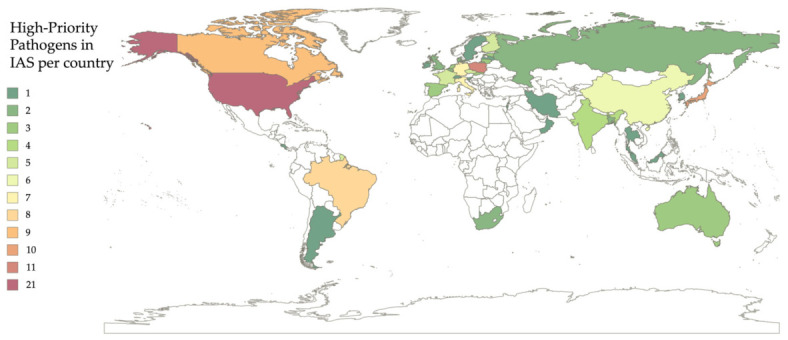
Geographical distribution of high-priority pathogen genera documented in IAS globally.

Lastly, the spatial analysis was extended to the global scale, revealing a comprehensive picture of the worldwide distribution of high-priority pathogens associated with the IAS. This geographical basis allowed for clear and rapid identification of the main hotspots where pathogen diversity and prevalence in non-native species for Europe are concentrated. These results clearly indicate that North America, particularly the United States, represents the main global hotspot, with the highest number of pathogen reports in the examined species, with a total of 21 high-priority pathogens across multiple IAS taxa. Europe, when considered as a unified entity encompassing all EU Member States and after data deduplication, emerges as a critical region, with 29 high-priority pathogens in IAS. Furthermore, Asia, especially the eastern and southern regions, including Japan and China, constitute the third largest hotspot. These findings provide the first integrated overview of the geographical distribution of high-priority pathogens associated with IAS at both EU and global scales (Figure 6).

The robustness of the HPI-IAS was evaluated for both the 2000–2026 and 2014–2026 temporal windows. In the 2000–2026 model, Monte Carlo simulations with randomized weight perturbations (±20–30%, renormalized) yielded a mean Spearman’s rank correlation of 0.996 (median = 0.997; 5th percentile = 0.987; 95th percentile = 1.000) and a mean Kendall’s tau of 0.973 (median = 0.977; 5th percentile = 0.932; 95th percentile = 1.000). The mean absolute rank shift across simulations was 0.33 positions (median = 0.31; 95th percentile = 0.75). Sensitivity to normalization showed very high agreement between min–max and z-score scaling (Spearman ρ = 0.998; Kendall τ = 0.977). Leave-one-out analyses produced Spearman correlations ranging from 0.962 to 0.979 and Kendall tau values ranging from 0.852 to 0.909, depending on the component removed.

For the 2014–2026 model, weight perturbation analyses resulted in a mean Spearman’s rank correlation of 0.995 (median = 0.997; 5th percentile = 0.986; 95th percentile = 0.999) and a mean Kendall’s tau of 0.969 (median = 0.977; 5th percentile = 0.926; 95th percentile = 0.994). The mean absolute rank shift was 0.36 positions (median = 0.34; 95th percentile = 0.88). Under alternative normalization schemes, rankings remained strongly correlated (Spearman ρ = 0.982; Kendall τ = 0.915). Leave-one-out procedures yielded Spearman correlations between 0.955 and 0.971 and Kendall tau values between 0.840 and 0.886.

## 4. Discussion

In this study, we conducted a systematic review of the literature to assess the current knowledge on the pathogens associated with IAS of EU concern and to identify major knowledge gaps in pathogen surveillance across Europe. However, the value of this work expands beyond a descriptive synthesis of pathogens in IAS. By integrating pathogen records with regulatory frameworks and by defining the HPI-IAS, we provide a comparative One Health framework that links invasion ecology with animal health, public health, and surveillance needs in EU Member States. In this way, our findings do not simply catalogue host–pathogen associations in these species but help identify which IAS and which geographic contexts may warrant greater surveillance priority at the wildlife–human–animal interface within the European invaded ecosystem. Specifically, focusing on 22 IAS of EU concern, among mammals, birds, reptiles, and amphibians, under the EU Reg. 1143/2014 and its implementing acts, our findings help identify IAS and EU Member States in which surveillance and risk mitigation efforts may be most urgently prioritized.

The results indicate that IAS are frequently associated with a wide range of infectious agents, including bacteria, viruses, endoparasites, protozoa and fungi, several of which are described here as high-priority pathogens. However, pathogen detection alone should not be interpreted as direct evidence of transmission, host competence, or actual public health impact. Rather, the occurrence of such pathogens in IAS highlights their potential epidemiological relevance and supports the need for further surveillance in matters of both public and animal health [249]. Although few studies have provided a similarly broad synthesis across IAS of EU concern, our findings are consistent with earlier works, suggesting that IAS may represent epidemiologically relevant interfaces in newly colonized ecosystems [30,249,250,251]. Multiple studies have suggested that IAS may function as reservoirs, maintenance hosts, amplifiers, or disseminators of infectious agents within the invaded ecosystem; thereby contributing to increased disease risk [30,252,253]. In recent years many interactions between IAS and zoonotic pathogens have been described, supporting their widespread involvement in pathogen transmission pathways [250]. Furthermore, IAS may introduce novel pathogens into the ecosystem or acquire local pathogens and contribute to their amplification, altering the host–pathogen dynamics and therefore the whole ecosystem [254,255,256], although the magnitude of these effects is likely to be pathogen-, host-, and context-dependent.

Although the present study did not directly assess the transmission dynamics or host competence, the frequent detection of priority pathogens in IAS suggests that these species may represent epidemiologically relevant interfaces for circulation and persistence of infectious agents outside their native habitat. However, this possibility has to be confirmed through species-specific studies addressing transmission, host competence, and exposure pathways. Therefore, IAS should be regarded as an integral component of a broader multi-host pathogen network rather than isolated epidemiological units, because pathogen transmission in ecological communities frequently involves interspecific spread and ecological connectivity across host species [257,258]. Indeed, our findings suggest that several widely established IAS in the EU are associated with multiple high-priority pathogens of relevance for both human and animal health. These species in the European continent occur in peri-urban, agricultural and natural environments, increasing the opportunity for pathogen transmission at the wildlife–livestock–human interface [259,260,261,262]. Additionally, the Host–Pathogen Influence Index (HPI-IAS) developed in this study provides a new tool to assess and compare the relative epidemiological pressure associated with IAS in the EU Member States.

Despite increasing interest, there is still a fragmented understanding of pathogen prevalence in IAS, especially across the EU. Research is disproportionately concentrated in a limited number of countries. It is sufficient to consider that, among the 27 member states of the EU, only 17 are represented in studies involving IAS high-priority pathogens. Moreover, some nations, such as Spain, Poland, Italy, and Germany, account for a substantial share of the available literature, suggesting a potentially significant geographic bias in research distribution. This imbalance in reporting distribution limits the comparability of pathogen identification across EU Member States and weakens our understanding of the true epidemiological patterns. In parallel with the uneven geographical distribution seen in Europe, the global literature on high-priority pathogens in IAS also reveals substantial geographic bias. Worldwide, only 20 countries are represented, which should raise concerns about the level of epidemiological surveillance of these species in their native environments. Therefore, apparent geographic differences in pathogen occurrence should be interpreted cautiously, as they may reflect uneven evidence availability rather than true differences in pathogen circulation. Most of the data originates from the Americas, where IAS native to this region, such as the raccoon, muskrat, coati, or gray squirrel, were associated with the highest amount of recorded pathogens. This association between pathogen richness and the hosts’ native region likely appears to be influenced by research intensity and, in part, more advanced wildlife surveillance systems, although intrinsic biological differences cannot be excluded [251]. Asia represents the second most documented region, whereas Africa and Oceania remain markedly underrepresented. This significant geographic imbalance likely reflects systemic barriers such as limited funding, language challenges in an English-dominated publishing system, and centralization of editorial boards in the global north [263], as well as inconsistencies in national surveillance priorities and reporting infrastructures [264]. As a result, global comparisons and risk assessments are inherently limited by this uneven data distribution. Moreover, little information is available from non-EU European countries. Although the number of records is limited, the geographical proximity to the EU makes this data highly relevant for regional surveillance. Invasive species may contribute to transboundary pathogen transmission, especially in regions sharing the same ecosystem, trading system, and the same migratory routes [26,250,265,266]. From a policy perspective, these findings highlight the importance of more harmonized cross-border surveillance, improved data sharing between EU and neighboring non-EU countries, and better integration of IAS monitoring into existing One Health and early warning frameworks.

Even though the EU Reg. 1143/2014 provides a common framework for contrasting IAS, the implementation remains fragmented. Each member state is responsible for implementing concrete actions in its territory, leading to potential disparities in surveillance capacity, based on know-how and funding, among others, as well as little cross-border collaboration. Because animal movements and pathogen transmission are not contained by national borders this collaboration needs to be implemented to collectively respond. Therefore, these challenges underscore the need for centralization of IAS enforcement actions, standardization of protocols, and reorganization of resources to ensure effective management and prevention of IAS-related pathogen spread across Europe [267].

Our results also revealed discrepancies in pathogen reporting for the species of concern, indicating a significant taxonomic bias in existing surveillance. Several species appear to be significantly underrepresented in terms of pathogen detection, and this applies mainly to non-mammalian species, like the eastern kingsnake and all the investigated birds. This underrepresentation may be attributed to a series of potentially overlapping factors, including limited surveillance on these species, an epidemiological role perceived as marginal, or a punctual investigation of pathogens in these species, a trend similarly highlighted previously [250]. This bias is clearly reflected in our dataset, with epidemiological attention concentrated on a limited number of well-studied mammalian species, in particular raccoons and raccoon dogs [260]. As a result, the apparent pathogen richness of these species likely reflects not only their biological relevance but also unequal research efforts. This interpretation aligns with broader zoonotic disease patterns, in which mammals, and rodents in particular, tend to receive disproportionate attention because of their recognized role in zoonotic systems [268]. Consequently, the lower pathogen richness observed in less-studied IAS should not be interpreted as evidence of lower epidemiological importance, but rather as an indicator of major knowledge gaps obscuring potentially relevant epidemiological associations.

In this context, protozoa and fungi appear to be among the least frequently reported pathogen groups associated with IAS. Overall, in all species analyzed, pathogenic fungi were identified in 26 different species across 11 IAS, comprising only 4.87% of the total pathogen species documented in alien species. While protozoan pathogens are reported in 66 species across 18 IAS, representing 13.98% of all recorded pathogens. On the basis of rational consideration, this apparent underrepresentation does not imply a true absence of fungal and protozoan pathogen presence in IAS but may instead reflect the historically lower prioritization of mycological surveillance in wildlife, as well as be the result of specialized diagnostic techniques not routinely applied in wildlife surveillance protocols or due to structural gaps in global surveillance systems [269,270,271]. In contrast, some fungal and protozoan species with zoonotic potential such as *Cryptococcus*, *Cryptosporidium*, and *Toxoplasma* have already been identified in IAS; due to their role in human disease, these species seem to have consistent inclusion and scientific interest in surveillance, representing an exceptional case despite their respective groups.

Lastly, very few longitudinal studies exist to understand how IAS-related pathogens evolve or spread over time and, furthermore, only a limited number of studies have begun to present information regarding host–pathogen interactions in IAS populations [254].

Much of the available data derived from the literature is obtained from cross-sectional or opportunistic samplings, giving only an epidemiological picture in these species. The lack of consistent prevalence over time undermines our ability to detect essential data from seasonal peaks, latency, temporal fluctuations, or long-term trends, ultimately not allowing us to understand how alien species adapt to new territories and local pathogens and how the pathogens they carry establish and evolve in the new ecosystem. Therefore, prolonged and continuous epidemiological surveillance is essential to assess how an IAS contributes epidemiologically within the new invaded habitat. For these reasons, epidemiological surveillance of IAS within the EU, and also globally, would lead to greater knowledge of the role these invasive species play both in their native environment and in the new territories they conquer. Enhanced surveillance would not only limit their negative health and environmental impacts but also preserve the feasibility of a possible and cost-effective eradication. As suggested by Koch et al. surveillance strategies become more efficient when logistical constraints are incorporated and spatial optimization is favored [271]. Therefore, in the EU, there is a pressing need for an optimized common diagnostic strategy, based on IAS of EU concern that pose a high risk of pathogen dissemination, particularly in known hotspots where these species are found. The epidemiological risk classifications developed in this study highlight the importance of an implementation of surveillance plans that not only target IAS in Europe but also their inherent biological risk of pathogen transmission and spread. By targeting IAS and their associated pathogens, it would be possible to have a complete European epidemiological picture that would allow for further developments in what has already been demonstrated, supporting early detection and rapid response efforts and the significant role that IAS play and extending knowledge beyond biodiversity loss or socio-economic effects, but more significantly at a health-related level for both animals and humans. These findings would underscore the emerging nature of these species whose negative potential is not yet fully understood, and therefore warrant scientific attention, especially in Europe where efforts in managing and contrasting IAS are already underway.

Our review confirms the substantial lack of current knowledge on pathogens affecting IAS. Despite the limited information available, it can be confirmed that IAS can harbor a wide range of pathogens. By integrating data from the scientific literature with global and EU frameworks it has been possible to identify the next pathogens of interest in these, often underestimated, species.

Our findings reveal that just a limited number of invasive species, including *Nyctereutes procyonoides*, *Procyon lotor*, *Trachemys scripta*, *Ondatra zibethicus* and *Sciurus carolinensis*, act as the main carriers of high-priority pathogens, precisely because of their zoonotic and epidemiological importance. This approach also made it possible to carry out a preliminary risk analysis of the presence of IAS and their associated pathogens within the EU, although the lack of data in each country remains a major source of bias. In this context, the HPI-IAS developed in this study provides a descriptive tool for assessing and comparing the epidemiological risk associated with IAS. By integrating data on IAS diversity, pathogen richness, and confirmed pathogen occurrence, the index helps identify countries in which ecological and epidemiological pressure associated with IAS may be higher, thereby supporting the prioritization of surveillance and management actions. However, because this analysis is descriptive and relies on secondary data, the results should be interpreted as indicative of relative surveillance needs rather than as quantitative risk estimates. Reported pathogen associations are influenced by differences in research effort, surveillance intensity, and methodological approaches across regions and host species. Widely distributed and extensively studied invasive species, such as the raccoon or raccoon dog, may appear associated with a higher pathogen richness partly due to greater research attention rather than intrinsic biological differences alone. Similarly, pathogen records derived from native or other introduced ranges do not necessarily imply equivalent pathogen presence or transmission dynamics within the EU, where ecological conditions and host–pathogen interactions may differ. The positive correlation observed between countries with greater IAS richness and those with high HPI-IAS values confirms the internal consistency of the index and highlights the ecological and surveillance heterogeneity in Europe. However, it is likely that countries with more effective monitoring systems also report a higher number of pathogen detections, partly inflating their apparent risk. The normalization of variables and the use of a composite indicator have mitigated this bias to some extent. Overall, this analytical approach integrates the ecological findings of the study, translating complex host–pathogen relationships into an interpretable framework on a continental scale. This provides a practical basis for identifying regions where surveillance and management actions need to be prioritized in order to limit the epidemiological impact of IAS. The robustness analyses demonstrate that the HPI-IAS exhibits strong structural stability across both long-term (2000–2026) and post-regulatory (2014–2026) temporal windows. Ranking correlations remained consistently high under weight perturbation, alternative normalization procedures, and leave-one-out tests, indicating that country positioning is not driven by arbitrary methodological choices. The slightly higher stability observed in the 2000–2026 model likely reflects the integrative effect of long-term invasion accumulation, which reduces the relative influence of short-term variability. In contrast, the 2014–2026 model, while still highly robust, displayed marginally greater sensitivity to normalization and component removal, suggesting that recent invasion dynamics may exert proportionally stronger effects when shorter temporal baselines are considered. Importantly, no single component dominated the index structure in either period, supporting the conceptual balance between invasion pressure, epidemiological relevance, pathogen infectivity potential, and observed pathogen evidence.

Collectively, these findings indicate that the HPI-IAS provides a stable and reproducible framework for assessing the host–pathogen influence of invasive alien species across European Union Member States, while remaining sufficiently sensitive to capture temporal shifts in invasion dynamics. The observed differences between the 2000–2026 and 2014–2026 HPI-IAS models provide important insights into the temporal dynamics of the host–pathogen interactions in IAS. The former model reflects the cumulative effect of IAS establishment, pathogen acquisition, and progressive ecological integration over longer time scales, capturing the gradual incorporation of IAS into the existing host–pathogen network. In contrast, the latter model is more sensitive to recent invasion events, newly detected pathogen associations, and increased post-regulatory surveillance efforts. For this reason, the 2000–2026 model may be considered more suitable for comparative epidemiological assessments and strategic surveillance planning, providing more stable and integrative representations of epidemiological patterns in the EU. Conversely, the 2014–2026 model may be more responsive to the dynamic nature of early invasion phases, during which pathogen acquisition, host adaptation, and surveillance detection are still evolving, although introducing greater susceptibility to short-term variability and surveillance-driven biases.

This study has several limitations that should be acknowledged. First, the analysis is based on published literature, which reflects heterogeneous research effort across regions, influenced by differences in surveillance capacity, scientific infrastructure, and funding availability. Consequently, countries and species that have been more extensively studied may appear to exhibit higher pathogen richness and epidemiological relevance due to detection bias rather than true biological differences. Second, pathogen records derived from native or other introduced ranges may not fully reflect the pathogen spectrum present within the EU, as ecological conditions, host density, and the presence of intermediate hosts may differ, potentially limiting pathogen establishment or transmission in newly invaded environments. Additionally, the identification of pathogens in IAS is largely based on presence data and does not necessarily reflect transmission dynamics, host competence, or actual impacts. Third, methodological variability across studies, including differences in diagnostic sensitivity, sampling strategies, and taxonomic focus, may influence pathogen detection probability. In particular, certain pathogen groups such as protozoa and parasites remain comparatively under characterized, and the detection of pathogen genetic material does not necessarily indicate active infection or epidemiological relevance. An additional limitation of this study is the exclusion of invertebrates and fish species, which are known to play important epidemiological roles, particularly within aquatic ecosystems. Their exclusion may have resulted in an incomplete representation of the overall epidemiological network associated with IAS. This exclusion reflects the specific focus of the study on terrestrial vertebrate hosts and should be considered when interpreting the results. Lastly, as highlighted in invasion biology research, estimating biological impact is inherently constrained by incomplete data availability, uncertainty regarding which ecological or epidemiological indicators are most appropriate for comparative assessments, and heterogeneity in the metrics used to quantify species performance and impacts [272]. Therefore, the HPI-IAS should be interpreted as a comparative epidemiological indicator reflecting relative host–pathogen influence and surveillance priorities, rather than as a direct quantitative measure of epidemiological risk. 

## 5. Conclusions

Our study confirmed that IAS are associated with various pathogens, many of which were identified as high-priority, with implications for both public and animal health. These findings reinforced the epidemiological significance of these species within the One Health framework and revealed both geographical and taxonomic knowledge gaps based on the available literature. Nevertheless, these results describe the presence and identification of pathogens and should not be interpreted to assess health impacts, transmission routes, or host–pathogen interactions. Stronger efforts are recommended at the European level to prioritize research in this field. Further information on biology, environmental characteristics, endogenous pathogens in a release area, and host/pathogen interactions, as well as the complex interactions between IAS and local species, will be essential to proceed with future risk analyses.

## Figures and Tables

**Table 1:** Priority pathogens according to the documents mentioned above.

Bacteria	Document	Viruses	Document	Parasites	Document
*Acinetobacter baumannii* (carbapenem resistant)	WHO Bacterial Priority Pathogens	*African swine fever virus*	Animal Health Law Reg. UE 2016/429	*Anisakis* spp.	Directive Zoonoses 2003/99/ec
*Bacillus anthracis*	Animal Health Law Reh. UE 2016/429	*Alphainfluenzavirus influenzae*	Directive Zoonoses 2003/99/EC	*Athina tumida*	Animal Health Law Reg. UE 2016/429
*Borrelia* spp.	Directive Zoonoses 2003/99/EC	*Alphainfluenzavirus influenzae*	Animal Health Law Reg. UE 2016/429	*Echinococcus multilocularis*	Animal Health Law Reg. UE 2016/429
*Brucella abortus*	Animal Health Law Reh. UE 2016/429	*Alphainfluenzavirus influenzae*	WHO Pathogen Prioritization	*Echinococcus* spp.	Directive Zoonoses 2003/99/ec
*Brucella melitentis*	Animal Health Law Reh. UE 2016/429	*Alphavirus chikungunya*	WHO Pathogen Prioritization	*Tenia solium*	Directive Zoonoses 2003/99/ec
*Brucella* spp.	Directive Zoonoses 2003/99/EC	*Alphavirus venezuelan*	Animal Health Law Reg. UE 2016/429	*Trichinella* spp.	Directive Zoonoses 2003/99/ec
*Brucella suis*	Animal Health Law Reh. UE 2016/429	*Aphtovirus vesiculae*	Animal Health Law Reg. UE 2016/429	*Tropilaelaps* spp.	Animal Health Law Reg. UE 2016/429
*Burholderia mallei*	Animal Health Law Reh. UE 2016/429	*Avian orthoavulavirus 1*	Animal Health Law Reg. UE 2016/429	*Varroa* spp.	Animal Health Law Reg. UE 2016/429
*Campylobacter* spp.	Directive Zoonoses 2003/99/EC	*Betacoronavirus cameli*, *B. erinacei*, *B. pipistrelli*, *B. tylonycteridis*	WHO Pathogen Prioritization	**Protozoa**	**Document**
*Chlamydia psittaci*	Directive Zoonoses 2003/99/EC	*Betacoronavirus pandemicum*	WHO Pathogen Prioritization	*Cryptosporidium* spp.	Directive Zoonoses 2003/99/ec
*Clostridium bolutinum*, *C. butyricum*, *C. barati*	Directive Zoonoses 2003/99/EC	Caliciviridae	Directive Zoonoses 2003/99/EC	*Sarcocystis* spp.	Directive Zoonoses 2003/99/ec
*Escherichia coli O157* (VTEC)	Directive Zoonoses 2003/99/EC	*Capripoxvirus lumpyskinpoc*	Animal Health Law Reg. UE 2016/429	*Toxoplasma* spp.	Directive Zoonoses 2003/99/ec
Enterobacteriales (3rd generation cephalosporin resistant and carbapenem resistant)	WHO Bacterial Priority Pathogens	*Deltaretrovirus bovleu*	Animal Health Law Reg. UE 2016/429	*Trypanosoma envasi*	Animal Health Law Reg. UE 2016/429
*Enterococcus faecium* (cancomycin resistant)	WHO Bacterial Priority Pathogens	*Hepatovirus ahepa*	Directive Zoonoses 2003/99/EC	*Trypanosoma equiperdum*	Animal Health Law Reg. UE 2016/429
*Francisella* spp.	Directive Zoonoses 2003/99/EC	*Lentivirus equinfane*	Animal Health Law Reg. UE 2016/429	**Fungi**	**Document**
*Haemophilus influenzae* (ampicillin resistant)	WHO Bacterial Priority Pathogens	*Lyssavirus rabies*	Directive Zoonoses 2003/99/EC	*Aspergillus fumigatus*	WHO Fungal Priority Pathogens
*Klebsiella pneumoniae*	WHO Pathogens Prioritization	*Mammarenavirus lassaense*	WHO Pathogen Prioritization	*Batrachochytrium salamandrivorans*	Animal Health Law Reg. UE 2016/429
*Leptospira* spp.	Directive Zoonoses 2003/99/EC	*Morbillivirus caprinae*	Animal Health Law Reg. UE 2016/429	*Candida albicans*	WHO Fungal Priority Pathogens
*Listeria monocytogenes*	Directive Zoonoses 2003/99/EC	*Morbillivirus pecoris*	Animal Health Law Reg. UE 2016/429	*Candida auris*	WHO Fungal Priority Pathogens
Mycobacterium bovis complex	Directive Zoonoses 2003/99/EC	*Orbivirus alphaequi*	Animal Health Law Reg. UE 2016/429	*Candida parapsilosis*	WHO Fungal Priority Pathogens
*Mycobacterium tuberculosis* (rifampicin resistant)	WHO Bacterial Priority Pathogens	*Orbivirus caerulinguae*	Animal Health Law Reg. UE 2016/429	*Candida tropicalis*	WHO Fungal Priority Pathogens
Mycobacterium tuberculosis complex	Animal Health Law Reh. UE 2016/429	*Orbivirus ruminantium*	Animal Health Law Reg. UE 2016/429	*Coccidioides* spp.	WHO Fungal Priority Pathogens
*Mycoplasma capricolum*	Animal Health Law Reh. UE 2016/429	*Orthoebolavirus sudanens*	WHO Pathogen Prioritization	*Cryptococcus neoformans*	WHO Fungal Priority Pathogens
*Mycoplasma mycoides* subsp. *Mycoides*	Animal Health Law Reh. UE 2016/429	*Orthoebolavirus zairense*	Animal Health Law Reg. UE 2016/429	Eumycetoma causative agents	WHO Fungal Priority Pathogens
*Neisseria gonorrhoeae* (3rd generation cephalosporin and/or fluoroquinolone resistant)	WHO Bacterial Priority Pathogens	*Orthoebolavirus zairense*	WHO Pathogen Prioritization	*Fusarium* spp.	WHO Fungal Priority Pathogens
*Pseudomonas aeruginosa* (carbapenem resistant)	WHO Bacterial Priority Pathogens	*Orthoflavivirus dengue*	WHO Pathogen Prioritization	*Histoplasma* spp.	WHO Fungal Priority Pathogens
*Salmonella enterica* subsp. Enterica serotype Enteridis, S. Typhimurium, S. Newport, S. Heidelberg, S. Javiana (fluoroquinolone resistant)	WHO Bacterial Priority Pathogens	*Orthoflavivirus flavi*	WHO Pathogen Prioritization	*Lomentospora prolificans*	WHO Fungal Priority Pathogens
*Salmonella enterica* subsp. Enterica serotype Enteridis, S. Typhimurium, S. Newport, S. Heidelberg, S. Javiana	WHO Pathogens Prioritization	*Orthoflavivirus japonicum*	Animal Health Law Reg. UE 2016/429	Mucorales	WHO Fungal Priority Pathogens
*Salmonella* spp.	Directive Zoonoses 2003/99/EC	*Orthoflavivirus nilense*	Animal Health Law Reg. UE 2016/429	*Nakaseomyces glabrata*	WHO Fungal Priority Pathogens
*Salmonella typhi* (fluoroquinolone resistant)	WHO Bacterial Priority Pathogens	*Orthoflavivirus ziakense*	WHO Pathogen Prioritization	*Paracoccidioides* spp.	WHO Fungal Priority Pathogens
*Shigella dysenteriae* serotype 1	WHO Pathogens Prioritization	*Orthohantavirus hantanense*	WHO Pathogen Prioritization	*Pichia kudriavzeveii*	WHO Fungal Priority Pathogens
*Shigella* spp. (fluroroquinolone resistant)	WHO Bacterial Priority Pathogens	*Orthomargburgvirus margburgense*	WHO Pathogen Prioritization	*Pneumocystis jirovecii*	WHO Fungal Priority Pathogens
*Staphylococcus aureus* (methicillin resistant)	WHO Bacterial Priority Pathogens	*Orthonairovirus haemorrgagiae*	WHO Pathogen Prioritization	*Scedosporium* spp.	WHO Fungal Priority Pathogens
*Streptococcus agalactiae* (penicillin resistant)	WHO Bacterial Priority Pathogens	*Orthopoxvirus monkeypox*	WHO Pathogen Prioritization	*Talaromyces mannaffei*	WHO Fungal Priority Pathogens
*Streptococcus pneumoniae* (macrolide resistant)	WHO Bacterial Priority Pathogens	*Pestivirus bovis*, *P. tauri*, *P. brazilense*	WHO Pathogen Prioritization		
*Streptococcus pyogenes* (macrolide resistant)	WHO Bacterial Priority Pathogens	*Pestivirus suis*	Animal Health Law Reg. UE 2016/429		
*Vibrio cholera* O139	WHO Pathogens Prioritization	*Phlebovirus riftense*	Animal Health Law Reg. UE 2016/429		
*Vibrio parahaemolyticus*, *V. vulnificus*, *V. alginolyticus*	Directive Zoonoses 2003/99/EC	*Togaviridae*, *Flaviviridae*, *Bunyaviridae*,	Directive Zoonoses 2003/99/EC		
*Yersinia pestis*	WHO Pathogens Prioritization	*Varicellovirus bovinealpha1*	Animal Health Law Reg. UE 2016/429		
*Yersinia* spp.	Directive Zoonoses 2003/99/EC	*Varicellovirus suidalpha 1*	Animal Health Law Reg. UE 2016/429		

**Table 2:** Results of the literature review: number of articles and pathogens reported for each IAS of European concern.

Host Species	N Articles	Observed Pathogens					
		Total	Bacteria	Virus	Fungi	Endoparasites	Protozoa
*Acridotheres tristis*	12	15	3	7	1	0	4
*Alopochen aegyptiacus*	6	5	2	2	0	0	1
*Axis axis*	10	7	3	2	0	0	2
*Callosciurus erythraeus*	12	5	0	1	1	1	2
*Callosciurus finlaysonii*	4	3	0	1	1	1	0
*Corvus splendens*	8	11	7	1	0	0	3
*Herpestes javanicus*	8	6	5	1	0	0	0
*Lampropeltis getula*	1	1	0	0	0	0	1
*Lithobates catesbeianus*	21	10	3	1	1	4	1
*Muntiacus reevesi*	8	5	2	1	0	0	2
*Myocastor coypus*	39	20	7	3	1	5	4
*Nasua nasua*	58	27	9	4	3	5	6
*Nyctereutes procyonoides*	80	88	15	12	3	48	10
*Ondatra zibethicus*	31	54	9	8	3	26	8
*Oxyura jamaicensis*	6	7	1	1	0	5	0
*Procyon lotor*	110	95	17	18	2	48	10
*Pycnonotus cafer*	2	1	0	0	0	0	1
*Sciurus carolinensis*	48	40	7	17	5	7	4
*Sciurus niger*	19	22	4	2	0	13	3
*Tamias sibiricus*	19	6	2	1	0	2	1
*Threskiornis aethiopicus*	4	3	2	1	0	0	0
*Trachemys scripta*	36	41	22	4	2	10	3
Total	541	472	120	88	23	175	66

**Table 7:** High-priority fungal species in IAS.

Fungi	Document	IAS Involved	Pathogen Species	Location EU	Location EXTRA EU
*Candida albicans*	WHO Fungal Priority Pathogens	**Mammals**: *Sciurus carolinensis*	*C. albicabs*	**Italy** (Cruciani et al. (2022) [106])	
*Cryptococcus gattii*	WHO Fungal Priority Pathogens	**Mammals**: *Sciurus carolinensis*	*C. gattii*		**Canada** (Duncan et al. (2006) [247])
*Cryptococcus neoformans*	WHO Fungal Priority Pathogens	**Mammals**: *Callosciurus finlaysonii*	*C. neoformans*	**Italy** (Iatta et al. (2015) [244])	
*Fusarium* spp.	WHO Fungal Priority Pathogens	**Reptiles**: *Trachemys scripta*	*F. falciforme*, *F. keratoplasticum*	**Spain** (Martínez-Ríos et al. (2022) [245])	
*Histoplasma* spp.	WHO Fungal Priority Pathogens	**Mammals**: *Nasua nasua*	*Histoplasma* spp.		**Brazil** (Costa et al. (1994) [246])
Eumycetoma causative agents (*Madurella* spp., *Falciformispora senegalensis*, *Curvularia lunata*, *Scedosporium* spp., *Zopfia rosatii*, *Acermonium* spp., *Fusarium* spp.)	WHO Fungal Priority Pathogens	**Reptiles**: *Trachemys scripta*	*F. falciforme*, *F. keratoplasticum*	**Spain** (Martínez-Ríos et al. (2022) [245])	

**Table 8:** High-priority pathogens in invasive alien species described in European countries.

Pathogen Species		*Callosciurus finlaysonii*	*Muntiacus reevesi*	*Sciurus carolinensis*	*Tamias sibiricus*	*Ondatra zibethicus*	*Trachemys scripta*	*Procyon lotor*	*Myocastor coypus*	*Nyctereutes procyonoides*
Bacteria	*Borrelia* spp.			1	3			3		4
	*Clostridium butyricum*						1			
	*Francisella tularensis*									1
	*Leptospira interrogans*					1	1	1	1	
	*Mycobacterium bovis*		1							
	*Salmonella enterica*						1			
Viruses	*Rabies virus*									1
	*West Nile virus*			1						
Fungi	*Candida albicans*			1						
	*Cryptococcus neoformans*	1								
	*Fusarium* spp.						2			
Endoparasites	*Echinococcus multilocularis*					1			1	1
	*Trichinella* spp.							1		4
Protozoa	*Cryptosporidium* spp.				1		1	1	1	1
	*Sarcocystis* spp.							1		
	*Toxoplasma gondii*					1			1	1

## Data Availability

The original contributions presented in this study are included in the article/Supplementary Material. Further inquiries can be directed to the corresponding author.

## Supplementary Materials

The following supporting information can be downloaded at: https://www.mdpi.com/article/10.3390/ani16091303/s1, Table S1. Host-Pathogen Influence index for Invasive Alien Species (HPI-IAS) calculated for each EU country in the interval 2000–2026. Using the presence of key invasive alien species, the potential epidemiological impact associated with the identified key species, and the occurrence of high-priority pathogens as variables. Construction of the Host-Pathogen Influence Index for Invasive Alien Species (HPI-IAS). Number of IAS = number of total IAS in the country; P_norm = biological pressure; Number of Key IAS = total number of Key IAS in the country; H_norm = normalized proportion of Key IAS in the country; Lo_row = observed pathogen load; Lo_norm = normalized observed pathogen load; S_norm = normalized spatial expansion in the country; P_star = Dynamic invasion pressure; Lp_epi_norm = normalized potential epidemiological burden. Table S2. Host-Pathogen Influence index for Invasive Alien Species (HPI-IAS) calculated for each EU country in the interval 2014–2026. Using the presence of key invasive alien species, the potential epidemiological impact associated with the identified key species, and the occurrence of high-priority pathogens as variables. Construction of the Host-Pathogen Influence Index for Invasive Alien Species (HPI-IAS). Number of IAS = number of total IAS in the country; P_norm = biological pressure; Number of Key IAS = total number of Key IAS in the country; H_norm = normalized proportion of Key IAS in the country; Lo_row = observed pathogen load; Lo_norm = normalized observed pathogen load; S_norm = normalized spatial expansion in the country; P_star = Dynamic invasion pressure; Lp_epi_norm = normalized potential epidemiological burden. Table S3. Distribution of IAS of Union concern across EU Member States during the period 2000–2026. W_2000 = spatial occurrence in 2000; W_2026 = spatial occurrence in 2026; G_c,s 2000–2026_ = proportional spatial expansion of species in country across the considered temporal windows; G_c_ = mean species-level spread index aggregated at the country level; S_norm= normalized spread of IAS per country. Table S4. Distribution of invasive alien species (IAS) of Union concern across EU Member States during the period 2014–2026. W_2014 = spatial occurrence in 2014; W_2026 = spatial occurrence in 2026; G_c,s 2014–2026_ = proportional spatial expansion of species in country across the considered temporal windows; G_c_ = mean species-level spread index aggregated at the country level; S_norm = normalized spread of IAS per country. Table S5. Pathogenicity assessment and epidemiological relevance criteria used for the high-priority pathogens in the HPI-IAS calculation. A_host = host breadth (specialist = 0, multi-host = 1, generalist = 2); B_transmission = transmission route complexity (complex/rare = 0, moderate =1, direct/environmental = 2); C_cycle = life-cycle structure (obligate indirect = 0, flexible indirect = 1, direct = 2); D One Health = One Health impact level (low = 0, high = 1); Esp = total epidemiological score for the pathogen; Lp_epi_raw = potential epidemiological burden of pathogen scores in IAS within a country; Lp_epi_norm = normalized potential epidemiological burden.
