# Supplementary material for: Invasive Alien Species of European Union Concern: A Systematic Review of High-Priority Pathogens in 22 Species in a One Health Framework
Source: Animals (Basel). 2026 Apr 23;16(9):1303. doi: 10.3390/ani16091303 (PMC13163108; doi:10.3390/ani16091303)
Supplement: Supplementary file 1 [file animals-16-01303-s001.zip › Supplementary Materials.pdf]

animals

MDPI

Supplementary Materials

1 Supplementary Tables

Table S1. Host-Pathogen Influence index for Invasive Alien Species (HPI-IAS) calculated for each EU country in the interval 2000-2026. Using the presence of key invasive alien species, the potential epidemiological impact associated with the identified key species, and the occurrence of high-priority pathogens as variables.

Construction of the Host-Pathogen Influence Index for Invasive Alien Species (HPI-IAS). Number of IAS = number of total IAS in the country; P_norm = biological pressure; Number of Key IAS = total number of Key IAS in the country; H_norm = normalized proportion of Key IAS in the country; Lo_row = observed pathogen load; Lo_norm = normalized observed pathogen load; S_norm = normalized spatial expansion in the country ; P_star = Dynamic invasion pressure; Lp_epi_norm = normalized potential epidemiological burden.

Table S2. Host-Pathogen Influence index for Invasive Alien Species (HPI-IAS) calculated for each EU country in the interval 2014-2026. Using the presence of key invasive alien species, the potential epidemiological impact associated with the identified key species, and the occurrence of high-priority pathogens as variables.

Construction of the Host-Pathogen Influence Index for Invasive Alien Species (HPI-IAS). Number of IAS = number of total IAS in the country; P_norm = biological pressure; Number of Key IAS = total number of Key IAS in the country; H_norm = normalized proportion of Key IAS in the country; Lo_row = observed pathogen load; Lo_norm = normalized observed pathogen load; S_norm = normalized spatial expansion in the country ; P_star = Dynamic invasion pressure; Lp_epi_norm = normalized potential epidemiological burden.

Table S3. Distribution of IAS of Union concern across EU Member States during the period 2000–2026. W_2000 = spatial occurrence in 2000; W_2026 = spatial occurrence in 2026; $G_{c,s\ 2000\text{-}2026}$= proportional spatial expansion of species in country across the considered temporal windows; G_c= mean species-level spread index aggregated at the country level; S_norm= normalized spread of IAS per country.

Table S4. Distribution of invasive alien species (IAS) of Union concern across EU Member States during the period 2014–2026. W_2014 = spatial occurrence in 2014; W_2026 = spatial occurrence in 2026; $G_{c,s\ 2014\text{-}2026}$= proportional spatial expansion of species in country across the considered temporal windows; G_c= mean species-level spread index aggregated at the country level; S_norm= normalized spread of IAS per country.

Table S5. Pathogenicity assessment and epidemiological relevance criteria used for the high-priority pathogens in the HPI-IAS calculation. A_host = host breadth (specialist = 0, multi-host = 1,

generalist = 2) ; B_transmission = transmission route complexity (complex/rare = 0, moderate =1, direct/environmental = 2); C_cycle = life-cycle structure (obligate indirect = 0, flexible indirect = 1, direct = 2); D One Health = One Health impact level (low = 0, high = 1); Esp = total epidemiological score for the pathogen; Lp_epi_raw = potential epidemiological burden of pathogen scores in IAS within a country; Lp_epi_norm = normalized potential epidemiological burden.

https://doi.org/10.3390/10.3390/ani16091303
